# A diet rich in high-glucoraphanin broccoli interacts with genotype to reduce discordance in plasma metabolite profiles by modulating mitochondrial function^1^,^2^,^3^

**DOI:** 10.3945/ajcn.113.065235

**Published:** 2013-08-14

**Authors:** Charlotte N Armah, Maria H Traka, Jack R Dainty, Marianne Defernez, Astrid Janssens, Wing Leung, Joanne F Doleman, John F Potter, Richard F Mithen

**Affiliations:** 1 From the Food and Health Programme, Institute of Food Research, Norwich Research Park, Norwich, United Kingdom (CNA, MHT, JRD, MD, AJ, WL, JFD, and RFM), and the Faculty of Medicine and Health Sciences, University of East Anglia, Norwich Research Park, Norwich, United Kingdom (JFP).

## Abstract

**Background:** Observational and experimental studies suggest that diets rich in cruciferous vegetables and glucosinolates may reduce the risk of cancer and cardiovascular disease (CVD).

**Objective:** We tested the hypothesis that a 12-wk dietary intervention with high-glucoraphanin (HG) broccoli would modify biomarkers of CVD risk and plasma metabolite profiles to a greater extent than interventions with standard broccoli or peas.

**Design:** Subjects were randomly assigned to consume 400 g standard broccoli, 400 g HG broccoli, or 400 g peas each week for 12 wk, with no other dietary restrictions. Biomarkers of CVD risk and 347 plasma metabolites were quantified before and after the intervention.

**Results:** No significant differences in the effects of the diets on biomarkers of CVD risk were found. Multivariate analyses of plasma metabolites identified 2 discrete phenotypic responses to diet in individuals within the HG broccoli arm, differentiated by single nucleotide polymorphisms associated with the *PAPOLG* gene. Univariate analysis showed effects of sex (*P* < 0.001), *PAPOLG* genotype (*P* < 0.001), and *PAPOLG* genotype × diet (*P* < 0.001) on the plasma metabolic profile. In the HG broccoli arm, the consequence of the intervention was to reduce variation in lipid and amino acid metabolites, tricarboxylic acid (TCA) cycle intermediates, and acylcarnitines between the 2 *PAPOLG* genotypes.

**Conclusions:** The metabolic changes observed with the HG broccoli diet are consistent with a rebalancing of anaplerotic and cataplerotic reactions and enhanced integration of fatty acid β-oxidation with TCA cycle activity. These modifications may contribute to the reduction in cancer risk associated with diets that are rich in cruciferous vegetables. This trial was registered at clinicaltrials.gov as NCT01114399.

## INTRODUCTION

Retrospective and prospective epidemiologic studies and associated meta-analyses have correlated diets that are rich in cruciferous vegetables, such as broccoli, with a reduced incidence and progression of cancer at several sites, including lung, stomach, colon, rectum, bladder, kidney, breast, and prostate (1–14). There is more limited evidence of a protective effect of cruciferous vegetables against cardiovascular disease (CVD)^4^ (15, 16).

Experimental studies with cell and animal models have provided evidence that isothiocyanates, derived from glucosinolates that specifically accumulate in these vegetables, may mediate a reduction in cancer and CVD risk through a multitude of mechanisms, the most prominent of which is induction of nuclear factor (erythroid-derived 2)-like 2 (Nrf2)–antioxidant response element–mediated phase II detoxification and antioxidant gene expression (17–20). Despite the evidence from cell and animal models, few data from human studies provide experimental evidence that diets rich in cruciferous vegetables may confer health benefits or provide an underlying mechanistic explanation for a reduction in either risk of cancer or CVD (21).

In animal models, Nrf2 has been shown to transcriptionally regulate many genes associated with central pathways of metabolism, including the regulation of genes involved in lipid and fatty acid synthesis, glycolysis, and the pentose phosphate pathway (22). Modulation of cell redox status is also likely to affect the activity of redox-sensitive enzymes such as aconitase and α-ketoglutarate dehydrogenase of the tricarboxylic acid (TCA) cycle (23). Thus, dietary intervention with Nfr2 inducers, such as isothiocyanates, may be expected to have effects on the plasma metabolite profile.

Mitochondrial dysfunction and metabolic perturbation are intimately associated with the development of chronic diseases such as type 2 diabetes, CVD, cancer, and neurologic disorders. The TCA cycle, the central hub of metabolism, not only functions in the generation of energy, but provides the precursors to many biosynthetic pathways, notably through the export of citrate leading to fatty acid and steroid biosynthesis and the export of oxaloacetate for gluconeogenesis. For maintenance of TCA cycle activity, these cataplerotic reactions need to be balanced by anaplerotic reactions that provide replacement TCA cycle intermediates (*see* Supplemental Figure S1 under “Supplemental data in the online issue). Suppression of TCA cycle activity, especially if associated with a high-fat diet, may also lead to dysfunctional integration of fatty acid β oxidation with the TCA cycle. This may lead to enhanced concentrations of acylcarnitines (the metabolic form in which acyl-CoAs is transported from the cytoplasm into the mitochondria) in the plasma (24–26). The occurrence of acylcarnitines in the plasma and urine has been associated with an inflammatory phenotype and as a risk factor for obesity and type 2 diabetes (24, 25) and kidney cancer (27).

We tested the hypotheses that a 12-wk dietary intervention with broccoli with an enhanced concentration of glucoraphanin (4-methylsulphinylbutyl glucosinolate), the precursor of the potent Nrf2 inducer sulforaphane, would have a greater effect on biomarkers of cardiovascular health and on plasma metabolic profiles than would an intervention with either standard broccoli or garden peas.

## SUBJECTS AND METHODS

### Participants

Men and women were enrolled into the study between January 2010 and February 2011. Volunteers with a 10-y CVD risk profile of between 10% and 20%, estimated by using the Joint British Societies 2 (JBS2) CVD risk assessor (28), were eligible to participate in the study. Those who were aged <50 y; had a history of stroke, myocardial infarction, or transient ischemic attack; had a BMI (in kg/m^2^) of <20 or >40, diagnosis of diabetes or a fasting glucose concentration of >6 mmol/L, blood pressure >160/90 mm Hg, or fasting cholesterol >8 mmol/L; taking any form of medication known to affect the cardiovascular system; or had a diagnosis of chronic kidney disease were excluded. Those who satisfied the above criteria entered the study after informed consent was obtained. Subjects were asked to continue any permitted prescribed medication for the duration of the trial and to inform the investigators of any changes in medication use. Volunteers taking certain supplements (eg, glucosamine and cod liver oil) were asked to discontinue use for ≥5 wk before starting the intervention. The study was approved by the Institute of Food Research Human Research Governance committee (IFR07/2009), Hertfordshire Research Committee (09/H031/96), and East Norfolk Waveney Research Governance Committee (2009IFR01). Because the study was adopted onto the NIH Research Clinical Research Network Portfolio, approval was also obtained from the NIH Research Coordinated System for Gaining National Health Service Permission (27377).

### Sample size

In the absence of previous studies that assessed changes in CVD risk profile, as determined by a Framingham-based model as an outcome measure within a broccoli dietary intervention study, the study was powered on anticipated changes in total serum cholesterol. Previous studies that had reported a reduction in total cholesterol after a broccoli sprout intervention (29) and mixed fruit and vegetable juice intervention (30) were used to provide an estimate of the potential effect of the broccoli intervention on cholesterol. To estimate the study size required, 3 assumptions were made: *1*) that the high-glucoraphanin (HG) broccoli cultivar would reduce total cholesterol by 1 mmol/L by the end of the intervention, the standard broccoli cultivar would reduce total cholesterol by 0.5 mmol/L, and the pea diet would not affect cholesterol concentrations; *2*) the mean baseline cholesterol concentration of each group was 6.0 mmol/L and the between-subject SD was 0.6 mmol/L; and *3*) the correlation between the baseline and final cholesterol concentration was 0.6. A minimum of 22 volunteers (in each group) would be required to detect a difference in mean serum total cholesterol of 0.5 mmol/L between volunteers fed the HG broccoli and the normal broccoli at a significance level of 0.05 for 80% power. A minimum of 10 volunteers would be required in the pea group to detect a difference in mean serum total cholesterol of 0.65 mmol/L between volunteers fed either the HG broccoli or the normal broccoli at a significance level of 0.05 for 80% power.

### Intervention

Male and female volunteers aged 50–77 y were recruited from within a 40-mile (64 km) radius of the city of Norwich, United Kingdom, through a combination of a volunteer database, general practitioner surgeries, newspaper advertisements, distributed flyers/poster, and word of mouth. Volunteers were recruited between January 2010 and February 2011 on the basis of a fasted (≥8 h) screening blood and urine sample and a completed health questionnaire; their risk score was calculated by using the JBS2 algorithm. A 12-wk randomized, 3-arm parallel study was then undertaken in men and women who, after screening, were judged to be at mild or moderate risk of CV based on the JBS2 risk estimator. Volunteers were required to consume 400 g HG broccoli, 400 g of a standard broccoli cultivar, or 400 g garden peas every week for 12 wk without any other dietary restrictions. The 2 broccoli genotypes, which looked identical, were coded by a third party, and their identity was unknown to the study investigators until completion of data collection. Volunteers were randomly assigned into the 3 intervention groups by using a minimization method, which reduced the baseline differences between the treatments, and each new volunteer was sequentially assigned to a group after the assignment of previous volunteers was taken into account.

The development and phenotypic analysis of HG broccoli has been reported previously (31). HG broccoli contains a Myb28 transcription factor, introgressed by conventional breeding from the wild species *Brassica villosa,* which results in enhanced assimilation of sulfate and channelling of the additional sulfur to glucoraphanin (31). Previous studies have reported the pharmacokinetics of sulforaphane metabolism in human volunteers after consumption of standard and HG broccoli (32).

The standard broccoli (cultivar Ironman) and the HG broccoli were grown and harvested by F Pettitt & Son (Boston Lincolnshire) with the use of standard agronomic procedures. Heads were harvested, floreted, blanched, and frozen as described previously by using standard commercial practice. After being processed, the HG broccoli contained 21.6 ± 1.60 μmol/g dry-weight glucoraphanin (4-methylsulphinylbutyl glucosinolate) and 4.5 ± 0.34 μmol/g dry-weight glucoiberin (3-methylsulphinylpropyl glucosinolate), whereas the standard broccoli contained 6.9 ± 0.44 μmol/g dry-weight glucoraphanin and 0.7 ± 0.33 μmol/g dry-weight glucoiberin. No differences in the concentrations of indole glucosinolate were found between the 2 types of broccoli. Frozen peas (Birds Eye) were purchased from a retailer. All vegetables were packaged into 100-g portions and stored frozen at −18°C. The frozen broccoli and peas were delivered to the volunteers in their homes every 3–6 wk or were collected by the volunteers from the study center. Volunteers prepared the broccoli cultivars from a frozen state by steaming for up to 5 min and the garden peas for up to 3 min. The volunteers were provided a steamer, written instructions, and a cooking demonstration.

### Biomarkers of cardiovascular health

The primary outcome measures were total serum cholesterol and 10-y CVD risk profile (calculated by using the JBS2 CVD risk-assessor model). Systolic and diastolic blood pressure, HDL cholesterol, LDL cholesterol, serum triglycerides, oxidized LDL, high-sensitivity C-reactive protein, pulse-wave velocity, and augmentation index were quantified as secondary outcome measures. Before providing a venous blood sample on the morning of the study days at the beginning and end of the intervention, the volunteers avoided caffeine and excessive exercise for 24 h and fasted for ≥8 hours. Lipids were analyzed by the Clinical Biochemistry Department of the Norfolk and Norwich University Hospital. An Abbott Architect C8000 analyzer was used to quantify lipid concentrations in both the pre- and postintervention blood samples. A cholesterol oxidase assay was used to assess total cholesterol, a cholesterol esterase assay was used to assess HDL cholesterol, and a glycerol kinase assay was used to assess triglycerides. The concentration of LDL cholesterol was calculated by using the Friedewald equation (33). After the blood samples were collected, an ambulatory blood pressure monitor (Spacelabs 90207; Spacelabs Health Care) was used to measure the volunteers’ blood pressure every 10 min for 1 h while they rested supine in a quiet, temperature-controlled room for 30 min before their arterial stiffness measurements. Carotid/femoral pulse-wave velocity was quantified with the Vicorder system (Skidmore Medical Ltd), and the augmentation index measured by using the SphygmoCor system of applanation tonometry (Atcor MedicalPty Ltd); the mean of 3 readings was calculated after correction for the heart rate. Oxidized LDL was quantified by ELISA from serum samples (10–1143-01; Mercodia AB), and human C-reactive protein was quantified by using a Quantikine assay from plasma samples (DCRP00; R & D Systems), according to the manufacturers’ instructions. The optical densities of the samples were read on a Biorad Benchmark Plus microplate reader within 30 min of the reactions being stopped. The CV% values of each assay across all plates read for C-reactive protein and oxidized LDL were 1.73% and 1.69%, respectively.

### Plasma metabolite profiles

Identification and relative quantification of metabolites in plasma were undertaken by Metabolon (www.metabolon.com) as previously described (34). A total of 347 metabolites were measured, spanning several relevant classes (amino acids, acylcarnitines, sphingomyelins, glycerophospholipids, carbohydrates, vitamins, lipids, nucleotides, peptides, xenobiotics, and steroids). The detection of the entire panel was carried out by using 3 mass spectrometry platforms: gas chromatography–mass spectrometry (GC-MS), liquid chromatography (LC)–MS optimized for positive ionization, and LC-MS optimized for negative ionization. Median process variability was <12% across all compounds measured in technical replicates. The resulting data were searched against standard libraries generated by Metabolon that included retention time, fragmentation patterns (GC-MS), *m*/*z*, preferred adducts and in-source fragments, and their associated MS/MS spectra (LC/MS) for all molecules in the libraries.

### Dietary intake analysis

Volunteers completed weekly tick sheets during the 12-wk intervention period to identify when the portions of vegetables were eaten. Every 2 wk, the volunteers were contacted by telephone and asked about adherence to the diet. A 7-d estimated food intake diet diary was completed by volunteers at baseline and after 10 wk by using household measures as an indication of portion size. Food intake from the diaries was entered into Diet Cruncher v1.6.1 software (www.waydownsouthsoftware.com/) and analyzed for differences in nutrient composition between the intervention groups at baseline and 10 wk after the start of the intervention.

### Single nucleotide polymorphism genotype analysis

Genomic DNA samples were isolated from the blood of 19 volunteers in the HG broccoli arm by using the QIAGEN DNeasy Blood & Tissue kit. DNA integrity was confirmed on an agarose gel, and samples were processed at the Genomics Centre at King's College London by using standard Affymetrix protocols. Briefly, 500–600 ng genomic DNA was used to generate NSP I and STY I restriction libraries from each sample and then amplified by PCR. Libraries were purified by using AMPure XP beads (Beckman Coulter), fragmented with DNase I digestion, and labeled with biotin. Hybridization was performed after Affymetrix recommendations onto Genome-Wide Human single nucleotide polymorphism (SNP) 6.0 arrays, and arrays were scanned by using the Affymetrix GeneChip Scanner. Data were analyzed in R/Bioconductor using the crlmm and genomewidesnp6Crlmm packages (35). All arrays had a signal-to-noise ratio >5. Of 906,600 SNPs genotyped, 680,530 had a confidence of >0.9 in all the samples and were further analyzed. To identify SNPs that would explain the 2 distinct metabolic profiles obtained from the volunteers we searched for SNP genotypes that were perfectly associated with the 2 phenotypic groups. Subsequently, DNA samples from all volunteers were genotyped for poly(A) polymerase gamma (PAPOLG; NM_022894.3) rs11687951, rs28459296, rs7579240, and *GSTM1* genotype by using predesigned Invitrogen TaqMan assays according to the manufacturers’ instructions.

### Analysis of CVD biomarkers

Each posttreatment response variable was analyzed for associations with sex, diet, *PAPOLG* rs7579240, *GSTM1,* and the interactions *PAPOLG* rs7579240 × diet, *GSTM1* × diet, sex × diet, sex × *PAPOLG* rs7579240, and sex × *GSTM1* by using an ANCOVA model in the R software package. The preintervention variable was used as a covariate in the model. Diagnostic checks were undertaken to ensure adherence to all model assumptions, including outlier tests and normality checks on the residuals.

### Principal component analyses of metabolite profiles

Principal component analysis (PCA) was conducted in Matlab (The Mathworks) by using the preintervention, postintervention, or ratio of post- to preintervention plasma metabolite data (48 individuals × 347 metabolites). PCA was also conducted on 2 subsets of data: the ratio of post- to preintervention data for the HG broccoli arm (19 individuals × 347 metabolites) and for the lipids (48 individuals × 141 metabolites). Xenobiotics (46 metabolites) and metabolites with an SD of 1 (resulting from data imputation of multiple missing values) were removed before all analyses.

### Associations between metabolites

To interpret the changes in metabolites, we sought to understand the interdependence or otherwise of each metabolite. We calculated regression coefficients and associated *P* values for all pairwise combinations of metabolites for which we had data (57,284 combinations) and corrected *P* values with a Bonferroni correction.

### Univariate analyses and Y scrambling

Data for individual metabolites were analyzed by using an ANCOVA model in the R software package. For each metabolite, the postintervention value of the log_2_ ion intensity was used as the response variable, and the preintervention value was used as a covariate. Sex, diet, and genotype were factor variables, and their main effects and interactions were estimated in the model. Because of the relatively small sample size, permutation tests (Y scrambling) were performed in R to assess the likelihood of the ANCOVA model results (36), whereby the response data were randomly shuffled, ANCOVA performed, and the results stored. This was repeated 1000 times for each metabolite. A similar approach was adopted for the analysis of metabolites within the HG broccoli arm. For the comparison of genotypes 1 and 2 with an unpaired *t* test, both before and after the intervention, all possible permutations of the genotype membership were used (75582 permutations). For changes in genotype 1 after the intervention, paired *t* tests were used whereby the pairs of pre- and postintervention values were kept, but for a random selection of pairs (ie, of individuals), the assignation of the 2 values to “pre” and “post” were swapped; this was done with all 2048 possible selections of pairs in the data set. A similar approach was used for genotype 2, with all 256 possible pair selections.

Y scrambling was used in preference to a Bonferroni correction, which requires variables (ie, metabolites) to be independent of each other, which we showed not to be the case. In addition, whereas a Bonferroni correction eliminates type I errors (ie, false-positive results), it results in a high frequency of type II errors (false-negative results) (37–39). Y scrambling prevents type II errors and provides a frequency distribution of potential type I errors that can be used to estimate the probability of the number of significant effects occurring by chance.

## RESULTS

### Volunteer recruitment

**FIGURE 1.**
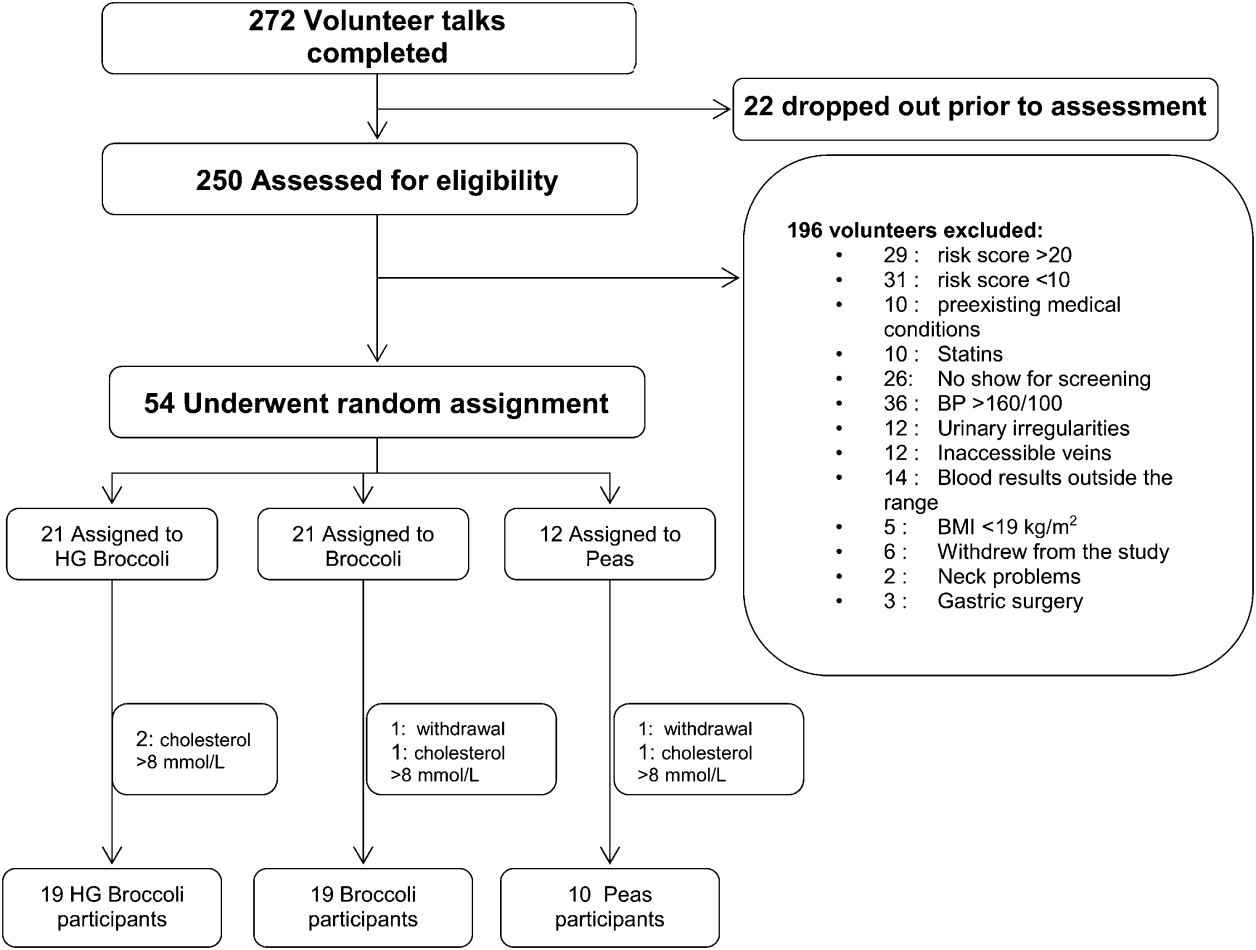
Flow diagram for volunteer recruitment. BP, blood pressure; HG, high glucoraphanin.

Of the 272 volunteers who attended introductory talks, 54 were recruited into a 12-wk randomized 3-arm parallel dietary intervention study. Of these, 4 were found to have a plasma concentration above the upper threshold for the study when sampled on the first study day, and 2 withdrew from the study. Forty-eight volunteers completed the study, 19 of whom were allocated to each of the broccoli arms and 10 to the pea arm. (**Figure 1**, **Table 1**). Forty-seven volunteers completed the weekly tick sheet, which indicated 100% compliance. The one volunteer who did not submit the tick sheet verbally confirmed full compliance. All the volunteers were nonsmokers, except for one male and one female in the standard broccoli arm and one male in the HG broccoli arm. All the diets were well received, and no adverse effects were reported. Nine of the 48 volunteers recruited into the study used a prescribed medication on an ad hoc basis, including ventolin, salbutamol, codydramol, and a cream for eczema. Volunteers were advised to inform us if they used their medication during the intervention, and none of them did.

**TABLE 1:** Baseline characteristics of the volunteers*^1^*

	Male			Female		
	Standard broccoli(*n* = 9)	HG broccoli(*n* = 10)	Peas(*n* = 5)	Standard broccoli(*n* = 10)	HG broccoli(*n* = 9)	Peas(*n* = 5)
Age (y)	57.3 ± 5.83	59.8 ± 7.28	62.0 ± 2.12	60.8 ± 5.31	63.8 ± 7.92	61.4 ± 2.51
BMI (kg/m^2^)	24.6 ± 3.17	25.8 ± 2.99	25.4 ± 2.79	26.0 ± 3.20	25.1 ± 4.48	26.4 ± 3.73
Waist (cm)	91.5 ± 10.39	94.1 ± 3.63	93.4 ± 6.39	83.8 ± 7.46	82.3 ± 12.38	85.6 ± 6.36
Systolic BP (mm Hg)	128 ± 6.2	128 ± 10.1	127 ± 10.3	137 ± 16.7	142 ± 11.7	132 ± 9.7
Diastolic BP (mm Hg)	78.0 ± 7.11	81.4 ± 8.59	80.4 ± 6.27	80.8 ± 9.81	85.8 ± 7.07	81.0 ± 3.32
Total cholesterol (mmol/L)	5.38 ± 0.96	5.12 ± 0.55	4.57 ± 0.62	6.54 ± 0.97	6.03 ± 0.72	5.57 ± 0.42
HDL cholesterol (mmol/L)	1.62 ± 0.54	1.47 ± 0.37	1.36 ± 0.11	1.94 ± 0.42	1.72 ± 0.27	1.44 ± 0.13
Triglycerides (mmol/L)	1.05 ± 0.36	1.19 ± 0.18	1.08 ± 0.47	1.59 ± 1.08	1.45 ± 0.65	1.36 ± 0.24
Glucose (mmol/L)	5.22 ± 0.34	5.32 ± 0.33	5.48 ± 0.32	5.25 ± 0.38	5.04 ± 0.47	4.72 ± 0.24
JBS2 CVD risk (%)	14.0 ± 0.03	14.0 ± 0.03	15.0 ± 0.03	13.0 ± 0.02	13.0 ± 0.03	13.0 ± 0.02

1 All values are means ± SDs. ANOVA showed no significant differences between sex or dietary arm for any variable. BP, blood pressure; CVD, cardiovascular disease; HG, high glucoraphanin; JBS2, Joint British Societies 2.

### Dietary intake analysis

No significant differences in habitual dietary intake were found between the 3 dietary groups or between the 2 assessment dates. Men had a significantly higher consumption of carbohydrates, protein, fat, sodium, chloride, and alcohol (*see* Supplemental Table S1 under “Supplemental data” in the online issue).

### Biomarkers of cardiovascular health

There was no evidence that diet, sex, or genotype had significant effects on biomarkers of cardiovascular health, with the exception of a possible effect of sex and genotype on pulse wave velocity (*see* Supplemental Table S2 and Figure S2 under “Supplemental data” in the online issue).

### Multivariate analysis of metabolic profiles

**FIGURE 2.**
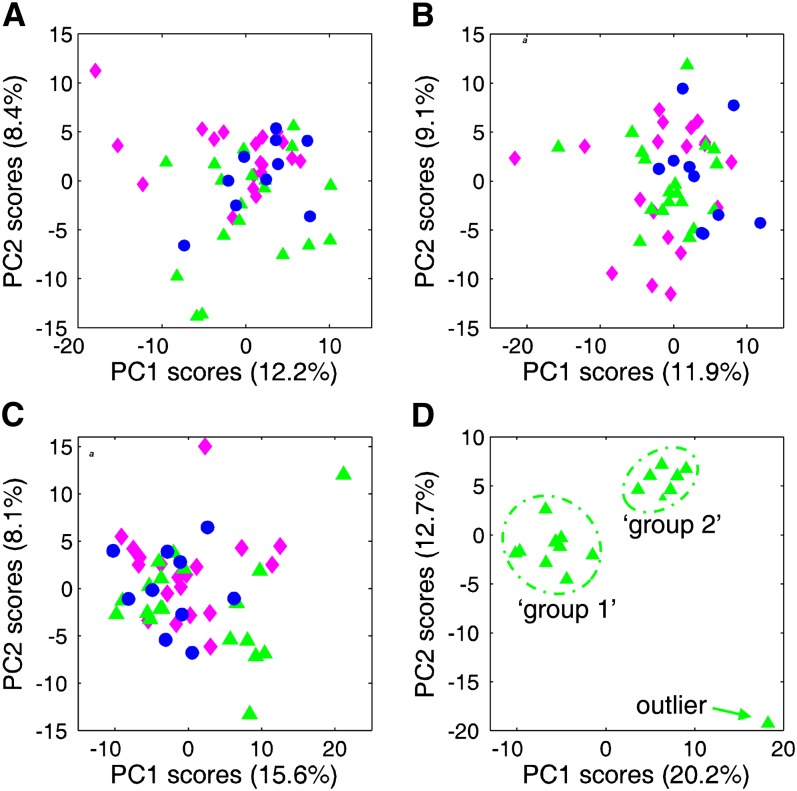
PCA of metabolomic data for all volunteers before the intervention (A; *n* = 48), all volunteers after the intervention (B; *n* = 48), the ratio of post- to preintervention data for all volunteers (C; *n* = 48), and the ratio of post- to preintervention data for the HG broccoli volunteers (D; *n* = 19). The green triangle represents the HG broccoli dietary arm, the pink diamond the standard broccoli arm, and the blue circle the pea dietary arm. HG, high glucoraphanin; PC, principal component; PCA, principal component analysis.

PCA of all metabolites (except xenobiotics) before (**Figure 2A**) and after (Figure 2B) the intervention did not indicate any discrete clustering of metabolic profiles associated with the dietary arm. There was an indication that, within the HG broccoli arm, there was less variability among individuals after the intervention than before because of a greater clustering of data points (Figure 2B). PCA of the ratio of post- to preintervention metabolites clearly indicated that the individuals with the HG broccoli arms were clustered within 2 distinct groups, with a single outlier (Figure 2C). The separation of the 2 groups was particularly apparent with a subsequent analysis of just the individuals in the HG broccoli arm (Figure 2D). Loading analyses of the PCA indicated that the major distinction between the 2 clusters was a result of variation in lipid and amino acid metabolites (*see* Supplemental Figure S3 under “Supplemental data” in the online issue). One volunteer in the HG intervention arm was an outlier. Further analysis with lipid metabolites only unambiguously associated this individual with phenotype group 2 (*see* Supplemental Figure S4 under “Supplemental data” in the online issue). Loading analysis identified long-chain and essential fatty acids as being the major class of lipid metabolites distinguishing the 2 phenotype groups (*see* Supplemental Figure S4 under “Supplemental data” in the online issue). These 2 well-differentiated groups within the HG broccoli arm were not differentiated by sex, age, BMI, or other demographic factors. Thus, we considered that the most likely reason for the differential response was a genetic polymorphism between these 2 groups.

### Genetic analysis of volunteers

We assayed 900,600 SNPs with the use of an Affymetrix SNP6 chip. Of these, only 3 SNPs, all associated with the *PAPOLG* gene, were perfectly associated with the 2 groups. One SNP was upstream of the start codon (rs 7579240), one in intron 3 (rs 28459296), and one in intron 8 (rs11687951). We genotyped all individuals in the study at each of these 3 SNPs with Taqman assays. Results from the HG volunteers were identical to those obtained from the Affymetrix SNP6 chip. Allele frequencies for each of the SNPs were similar to those previously reported, and genotypes were in Hardy-Weinberg equilibrium (*see* Supplemental Table S3 under “Supplemental data” in the online issue). All individuals in phenotype group 1 (Figure 2D) were homozygous for the major allele at each of the 3 SNP genotypes (genotype 1), whereas all individuals in phenotype 2 were either heterozygous or homozygous for the minor allele (genotype 2).

### Associations between metabolites

Before conducting the univariate analyses, we sought to understand the interdependence of pairs of metabolites. Of the 57,284 combinations, 3683 had correlation coefficients ≥0.5, whereas only 4 had a correlation coefficient ≤−0.5. Fatty acids tended to be highly correlated with each, as were steroid metabolites. Palmitoylcarnitine and oleolylcarinitine were correlated with lysolipids; 18.7% of all possible pairwise regressions analyses had an uncorrected *P* value of ≤0.05, which fell to 2.1% after a Bonferroni correction, which is likely to underestimate the true number of significant correlations because of a probable high frequency of type II errors, as previously discussed.

### Univariate analysis of metabolic profiles

The metabolic data were analyzed to take into consideration the effects of sex, diet, and the *PAPOLG* genotype and 2-factor interactions between these variables. To determine the robustness of the data and the expected number of false-positive results, the analyses were also undertaken after each of 1000 permutations of the data sets (Y scrambling). The results are summarized in **Table 2**, and full details are provided elsewhere (*see* Supplemental Table S4 under “Supplemental data” in the online issue). Forty-four metabolites were significantly associated with sex [median number of metabolites after Y scrambling = 20, *P* (44 metabolites) < 0.001]. As would be expected, men had higher concentrations of male-associated steroid hormones and higher concentrations of creatinine and branched-chain amino acid metabolites, both of which are associated with the higher amount of skeletal muscle found in men than in women. Women had higher levels of saccharin and benzoates and certain carbohydrate metabolites. Seventeen metabolites were significantly associated with diet. Because the median number of false-positive values occurring by chance is itself 17 (Table 2), we concluded that diet cannot be considered to have a significant effect on the metabolite profile. Fifty metabolites were significantly associated with the *PAPOLG* genotype [median number of false-positive values = 20, *P* (50 metabolites) < 0.001]. Most of these, which comprised lipid and amino acid metabolites—which confirmed the PCA—were significantly higher in genotype 2 than in genotype 1, with the notable exception of methionine and tyrosine, which were higher in genotype 1. In addition to lipid and amino acid metabolites, genotype had a significant effect on the level of the cofactor flavin adenine dinucleotide (FAD), which was significantly higher in genotype 2 than in genotype 1 (*P* < 0.001). Thirty-nine metabolites were significantly altered through a diet × genotype interaction [median number = 20, *P* (39 metabolites) <0.001], which were predominantly lipid and amino acid metabolites. The PCA (Figures 2C and D) indicated that the most apparent diet × genotype effects occurred in the HG broccoli arm, and this is explored in greater detail below.

**TABLE 2:** The number of metabolites that were significantly different by sex, diet, and *PAPOLG* genotype and the 2-factor interactions (*n* = 48)*^1^*

Pathway*^2^*	Sex	Diet	*PAPOLG* genotype	Sex × diet	Sex × gene	Diet × gene
Amino acids (85)	11*^2^*,*^3^*	3	8	1	2	4
Carbohydrates (23)	9	0	3	2	1	0
Cofactors and vitamins (15)	3	1	4	0	1	0
Energy (9)	1	0	1	1	0	1
Lipids (141)	15	7	29	6	6	31
Nucleotides (14)	1	2	3	0	0	2
Peptides (14)	0	1	0	0	0	0
Xenobiotics (46)	4	3	1	6	0	1
Total (347)	44	17	50	16	10	39
Median number after Y scrambling*^3^*	20	17	20	20	20	20
*^4^*	<0.001	NS	<0.001	NS	<0.001	<0.001

1 All values are the number of metabolites within each class that reached the achieved statistical threshold (*P* < 0.05). Details of the metabolites and *P* values are provided elsewhere (*see* Supplemental Table 4 under “Supplemental data” in the online issue). ANCOVA was used to analyze differences in metabolites between volunteers. For each metabolite, the postintervention value of the log_2_ ion intensity was used as the response variable, and the preintervention value was used as a covariate. Sex, diet, and genotype were factor variables, and their main effects and interaction were estimated in the model.

2 Numbers in parentheses are the total number of metabolites within each class.

3 The values were derived after 1000 permutations of the data set.

4 The probability that the total number of metabolites found to be different would have occurred by chance, estimated from the frequency distribution of metabolites that reached the statistical threshold (*P* < 0.05) after 1000 permutations of the data set.

### Analysis of metabolic profiles within the HG broccoli arm

**FIGURE 3.**
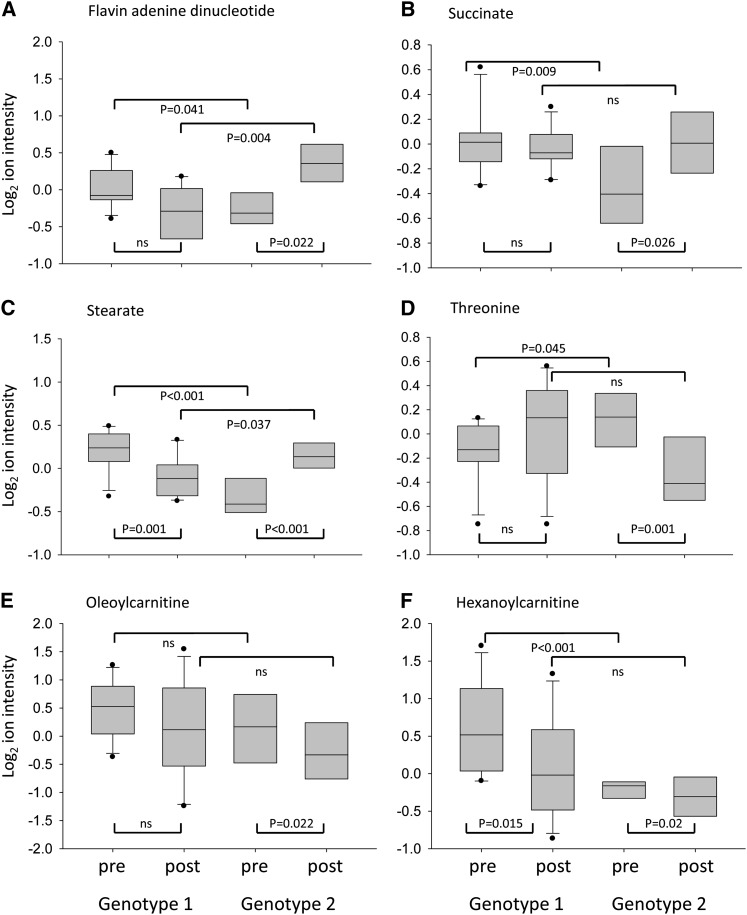
Box plots of changes in representative examples of metabolites within the high-glucoraphanin broccoli intervention arm. A: Flavin adenine dinucleotide. B: Succinate, as an example of a tricarboxylic acid intermediate. Malate and fumarate have a similar genotype × diet interaction. C: Stearate, as an example of a fatty acid. Approximately 50 lipid metabolites and a smaller number of amino acid metabolites, such as 3-methyl-2-oxybutyrate, show a similar pattern. D: Threonine, as an example of an amino acid. Phenylalanine, tryptophan, methionine, histidine, and glutamine had a similar pattern. E: Oleoylcarnitine as an example of acylcarnitines. F: Hexanoylcarnitine as an example of acylcarnitines. post, after intervention; pre, before intervention.

Before the intervention, there were 66 metabolites [median number of false-positive values after Y scrambling = 15, *P* (66 metabolites) <0.0001] that were significantly different between the 2 *PAPOLG* genotypes in the volunteers within the HG broccoli arm, of which 8 were acylcarnitines and 32 were other lipids [**Table 3**; full details are provided elsewhere (*see* Supplemental Table S5 under “Supplemental data” in the online issue)]. All of these metabolites, which included 3 TCA cycle intermediates (succinate, malate, and fumarate) and the cofactor FAD, were significantly higher in genotype 1 than in genotype 2, with the exception of the amino acids threonine, tryptophan, and phenylalanine, which were higher in genotype 2 than in genotype 1 (**Figure 3**; *see* Supplemental Table S5 under “Supplemental data” in the online issue). After the intervention, the differences between the concentrations of lipid and amino acids had either been eliminated or reversed. Thus, of the 38 metabolites that were now significantly different between the 2 genotypes, 23 lipids and FAD were now higher in genotype 2 than in genotype 1 (Table 3), a reverse of the previous pattern. The differences in TCA cycle intermediates between the 2 genotypes had been eliminated (Table 3, Figure 3). Concentrations of acylcarnitines were reduced in both genotypes after the dietary intervention, which eliminated any differences that occurred before the intervention (Table 3). The changes in relative metabolite concentrations between the 2 genotypes were a result of contrasting effects of the HG broccoli diet. A reduction in the concentration of lipid and amino acid metabolites was observed in genotype 1, whereas an increase was observed in genotype 2 (**Table 4**). This contrasting effect of diet was not true for acylcarnitines, for which there were reductions in both genotypes. Significant reductions in hexanoylcarnitine, octanoylcarnitine, and laurylcarnitine were observed in genotype 1, whereas significant reductions in palmitoylcarnitine and oleoylcarnitine were observed in genotype 2 (Table 4, Figure 3; *see* Supplemental Table S5 under “Supplemental data” in the online issue).

**TABLE 3:** The number of metabolites that were significantly different between *PAPOLG* genotypes 1 and 2 before and after the intervention in volunteers within the high-glucoraphanin broccoli arm (*n* = 19)*^1^*

	Before intervention		After intervention	
Pathway*^2^*	Higher in genotype 1	Higher in genotype 2	Higher in genotype 1	Higher in genotype 2
Amino acids (85)	8	3	1	5
Carbohydrates (23)	1	0	0	0
Cofactors and vitamins (15)	4	0	1	1
Energy (9)	3	0	0	0
Acylcarnitines (12)	8	0	0	0
Lipids (129)	32	0	0	23
Nucleotides (14)	2	0	1	3
Peptides (14)	0	1	0	0
Xenobiotics (46)	4	0	1	1
Total (347)	66		37	
Median number after Y scrambling*^3^*	15		15	
*^4^*	<0.0001		<0.02	

1 All values are the number of metabolites within each class that reached the achieved statistical threshold (*P* < 0.05). Details of the metabolites and *P* values are provided elsewhere (*see* Supplemental Table S5 under “Supplemental data” in the online issue). *t* Tests were used to analyze differences in the log_2_ ion intensity of each metabolite between the genotypes before and after the intervention.

2 Numbers in parentheses are the total number of metabolites within each class.

3 The values were derived after 75,528 permutations of the data set.

4 The probability that the total number of metabolites found to be different would have occurred by chance, estimated from the frequency distribution of metabolites that reached the statistical threshold (*P* < 0.05) after 75,528 permutations of the data set.

**TABLE 4:** The number of metabolites that were different before and after the *PAPOLG* genotypes 1 and 2 intervention in volunteers within the high-glucoraphanin broccoli arm (*n* = 19)*^1^*

	Genotype 1		Genotype 2	
Pathway*^2^*	Metabolites decreased by intervention	Metabolites increased by intervention	Metabolites decreased by intervention	Metabolites increased by intervention
Amino acids (85)	3	1	7	10
Carbohydrates (23)	0	0	0	0
Cofactors and vitamins (15)	1	0	0	2
Energy (9)	0	0	0	1
Acylcarnitines (12)	3	0	2	0
Lipids (129)	54	0	1	51
Nucleotides (14)	4	0	0	3
Peptides (14)	0	0	0	1
Xenobiotics (46)	0	0	2	2
Total (347)	66		82	
Median number after Y scrambling	14*^3^*		11*^4^*	
*^5^*	<0.002		<0.004	

1 All values are the number of metabolites within each class that reached the achieved statistical threshold (*P* < 0.05). Details of the metabolites and *P* values are provided elsewhere (*see* Supplemental Table S5 under “Supplemental data” in the online issue). *t* Tests were used to analyze differences in the log_2_ ion intensity of each metabolite within each genotype before and after the intervention.

2 Numbers in parentheses are the total number of metabolites within each class.

3 The value was derived after 2048 permutations of the data set.

4 The value was derived after 256 permutations of the data set.

5 The probability that the total number of metabolites found to be different would have occurred by chance, estimated from the frequency distribution of metabolites that reached the statistical threshold (*P* < 0.05) after permutations of the data set.

## DISCUSSION

We tested the hypothesis that a dietary intervention with HG broccoli would modify biomarkers of cardiovascular health and plasma metabolite profile to a greater extent than an intervention with standard broccoli or peas. We obtained no evidence of a differential effect of the HG broccoli diet on total, LDL, or HLD cholesterol; 10-y CVD risk; systolic or diastolic blood pressure; or other biomarkers of cardiovascular health compared with the other dietary arms (*see* Supplemental Table S1 and Figure S2 under “Supplemental data” in the online issue). These results do not support previous reports that broccoli reduces cholesterol concentrations (29) or data obtained from rodents studies extrapolated to humans (20). In contrast, we obtained evidence that diet interacted with genotype to result in significant changes in the plasma metabolite profile (Table 2).

The first indication that the effect of diet was to reduce metabolic variation among volunteers in the HG broccoli arm was the greater clustering observed in the postintervention PCA compared with preintervention PCA (Figure 2, A and B). Subsequent analysis of the ratio of metabolites before and after the intervention unambiguously identified 2 phenotypic responses to diet within the HG broccoli arm (Figure 2, C and D). Because these 2 phenotypic groups were not differentiated by any demographic factors, we postulated that it was a result of a genetic difference. Global analysis of SNPs identified genetic polymorphisms associated with the *PAPOLG* gene.

*PAPOLG* is a poorly characterized gene that is likely to be a member of the poly(A) polymerase family, which catalyses template-independent extension of the 3′ end of a DNA/RNA strand (40). The product of this gene, hsPAPγ, has been shown to interact with 4 proteins: DEAH (Asp-Glu-Ala-His) box polypeptide 29, leucine-zipper-like transcription regulator 1, nuclear transcription factor Y, and pterin-4 alpha-carbinolamine dehydratase (41); the latter of which is associated with activity of the transcription factor hepatocyte nuclear factor 1, which regulates a network of hepatic genes (42).

Univariate analyses, with Y scrambling to estimate the frequency of type 2 errors, demonstrated a significant effect of sex, *PAPOLG* genotype and a *PAPOLG* genotype × diet interaction on metabolic profiles (Table 2). A potentially important difference between the 2 genotypes was in the level of FAD, which after the intervention was higher in genotype 2 than genotype 1. FAD is a redox cofactor associated with several important reactions in metabolism, notably in fatty acid oxidation and glycolysis. Moreover, FAD modulates the activity of lysine-specific demethylase-1 (LSD1), which, through epigenetic regulation, fine tunes the expression of genes associated with mitochondrial metabolism and energy expenditure (43). The data we obtained are consistent with the regulatory effect of FAD; when FAD levels are relatively high, there is down-regulation of expression of genes in mitochondrial respiration and enhanced export of citrate from the TCA cycle, which results in elevated concentrations of fatty acids, lysolipids, and steroids. To compensate for the export of citrate, there would need to be amino acid anaplerosis, resulting in a decrease in primary amino acids in plasma and an increase in certain amino acid metabolites.

An inverse association of plasma amino acids and fatty acids was previously observed in 2 groups of lactating Holstein cows; contrasting metabolic strategies were used in that study to achieve similar levels of milk production. One group of cows had relatively high concentrations of plasma fatty acids but low concentration of plasma amino acids compared with a second group, and this difference was reflected in their respective levels of hepatic fat and different levels of expression of enzymes involved in hepatic fatty acid and amino acid metabolism (44). Furthermore, the cows that had higher concentrations of plasma fatty acids also had evidence of impaired defenses against oxidative stress (44), which confirmed other reports of an association between plasma fatty acids and markers of oxidative stress (44, 45) and emphasized the potential metabolic effects of Nrf2-inducers, such as sulforaphane.

Thus, in support of our hypothesis, the most striking effect of diet on metabolic profiles was within the volunteers who consumed the HG broccoli diet (Table 3; *see* Supplemental Table S5 under “Supplemental data” in the online issue). Results of an analysis of metabolite profiles before the intervention were consistent with the FAD-LSD1 hypothesis, ie, the dietary intervention enhanced the difference in the level of FAD between the 2 genotypes (Figure 3). As a consequence, the differences in the concentrations of lipid and amino acid metabolites observed before the study were either eliminated or reversed (Table 3). In addition, there were significantly higher concentrations of 8 acylcarnitines in genotype 1, which indicated a dysfunctional integration between fatty acid β oxidation, amino acid catabolism, and TCA cycle activity. After the interventions, the concentrations of acylcarnitines were reduced in both genotypes, and all differences in acylcarnitines between genotypes were eliminated (Figure 3), which suggested a better integration of fatty acid catabolism and TCA cycle among all volunteers. It is also notable that, before the intervention, significant discordance in concentrations of succinate, fumarate, and malate were found between the 2 genotypes, which was eliminated after the dietary intervention (Table 3, Figure 3). Thus, the overall effect of the intervention appeared to be a normalization of TCA cycle intermediates and metabolism, suggested by the reduction in acylcarnitines, and a balancing of cataplerotic and anaplerotic reactions.

Lower concentrations of urinary acylcarnitines have previously been reported to occur after a 2 wk intervention with a diet rich in cruciferous vegetables, citrus, and soy compared with a diet free of fruit and vegetables (46). Moreover, dietary intakes of coffee and garlic, both of which are associated with a reduced risk of chronic disease (47, 48) and are known to induce Nrf2-mediated transcription in model systems (49, 50), have been negatively correlated with plasma concentrations of acylcarnitines (51). These studies suggest a more generic mechanism by which diets rich in bioactive phytochemicals that induce Nrf2 gene expression may reduce the risk of chronic disease.

Further studies are required to elucidate how the HG broccoli diet mediates metabolism through a putative interaction with the *PAPOLG* genotype. The results we obtained are consistent with the FAD-LSD1 model for the control of metabolism. FAD is synthesized from dietary riboflavin through the activity of 2 enzymes: riboflavin kinase and *FLAD1* synthase. The latter of these uses ATP to catalyze the adenylation of flavin mononucleotide to form FAD. The putative activity of *PAPOLG* is to use ATP in adenylation reactions. Although there is no homology between *FLAD1* and *PAPOLG,* it is possible that there may be competition for substrate so that genetic differences in the *PAPOLG* gene may affect FAD levels. Note that modulation of LSD1 activity is a therapeutic target for prostate and other cancers (52).

The HG diet may also affect metabolism through additional multiple and complex interactions with redox sensitive regulatory proteins, such as phosphatase and tensin homolog and protein tyrosine phosphatase 1B, and other redox sensitive enzymes, such as those of the TCA cycle, that together affect metabolism. It is pertinent to note that sulforaphane, the isothiocyanate derived from glucoraphanin, can have both oxidant and antioxidant effects which may result in divergent physiologic consequences (53, 54).

In conclusion, we consider that this study provides an important and original insight into how diets rich in cruciferous vegetables may be able to reduce the risk of cancer. It also suggests that this protective effect is mediated by glucosinolates. As we age, increasing levels of mitochondrial dysfunction, possibly as a result of greater oxidative stress, leads to metabolic perturbations and an imbalance between the anaplerotic and cataplerotic reactions that are necessary to maintain the optimum balance between energy generation and the synthesis of fatty acids and other metabolites required for the maintenance of health. The nature of these changes within an individual depends on diet, physical activity, and genotype. A diet rich in cruciferous vegetables effectively retunes our metabolism by rebalancing anaplerosis and cataplerosis and restoring metabolic homeostasis. In this manner, cruciferous vegetables may be able to reduce the risk of many chronic diseases associated with aging.

## Supplementary Material

Author Video

Supplemental data

## Footnotes

4 Abbreviations used: CVD, cardiovascular disease; FAD, flavin adenine dinucleotide; GC, gas chromatography; HG, high glucoraphanin; JBS2, Joint British Societies 2; LC, liquid chromatography; LSD1, lysine-specific demethylase-1; MS, mass spectrometry; Nrf2, nuclear factor (erythroid-derived 2)-like 2; PAPOLG, poly(A) polymerase gamma; PCA, principal component analysis; SNP, single nucleotide polymorphism; TCA, tricarboxylic acid.
