# Supplementary material for: A diet rich in high-glucoraphanin broccoli interacts with genotype to reduce discordance in plasma metabolite profiles by modulating mitochondrial function1
Source: Am J Clin Nutr. 2013 Aug 14;98(3):712–22. doi: 10.3945/ajcn.113.065235 (PMC3743733; doi:10.3945/ajcn.113.065235)
Supplement: Author Video [file supp_98_3_712__index.html]

Supplemental data 

# A diet rich in high-glucoraphanin broccoli interacts with genotype to reduce discordance in plasma metabolite profiles by modulating mitochondrial function

## Supplemental data

**Files in this Data Supplement:**

- Supplemental data - Figures 1-4
- Supplemental data - Tables 1-5
