# Supplementary material for: A diet rich in high-glucoraphanin broccoli interacts with genotype to reduce discordance in plasma metabolite profiles by modulating mitochondrial function1
Source: Am J Clin Nutr. 2013 Aug 14;98(3):712–22. doi: 10.3945/ajcn.113.065235 (PMC3743733; doi:10.3945/ajcn.113.065235)
Supplement: Supplemental data [file 98.3.712_ajcn065235SupplementaryData1.pdf]

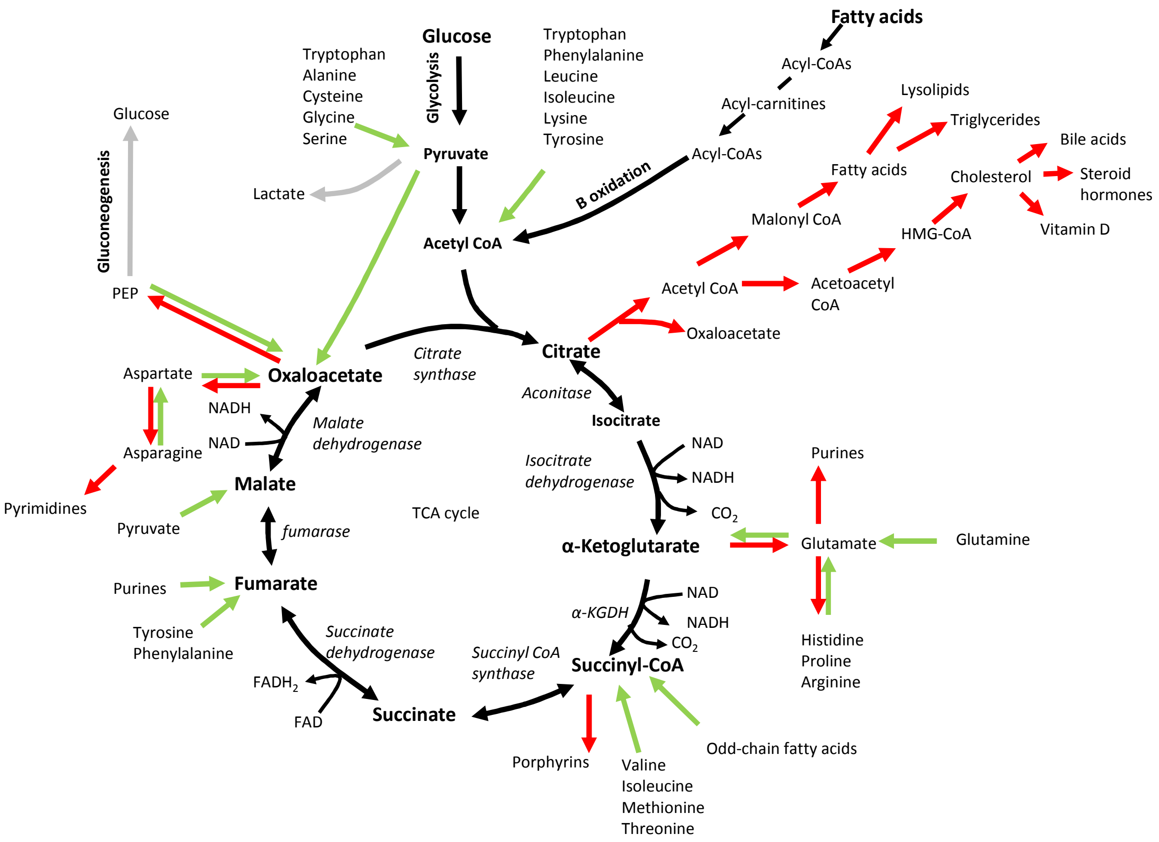

SUPPLEMENTAL FIGURE S1. Tricarboxylic acid cycle with anaplerotic reactions (green) and cataplerotic reactions (red)

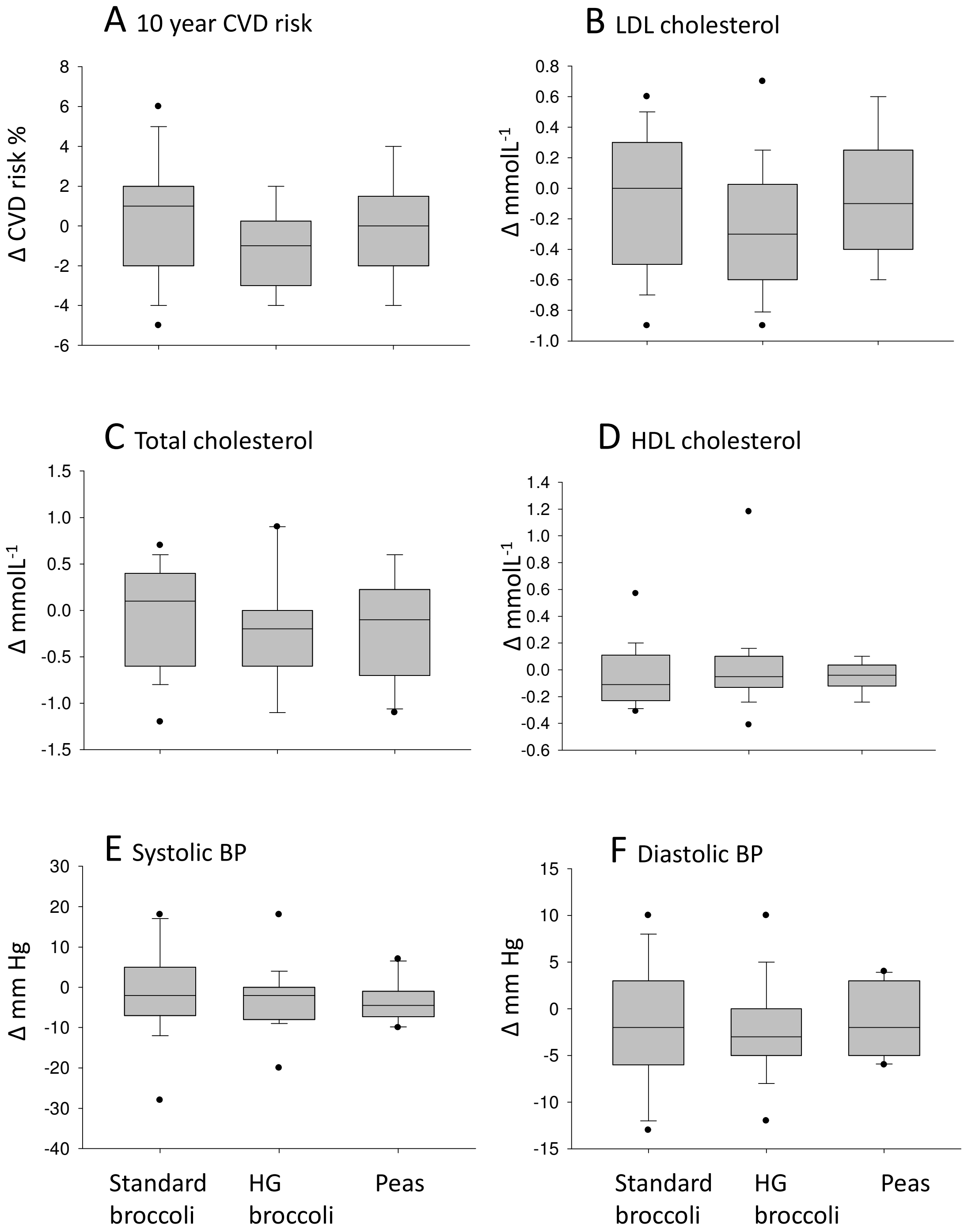
A 10 year CVD risk
Δ CVD risk %
8
6
4
2
0
-2
-4
-6
B LDL cholesterol
Δ mmolL-1
0.8
0.6
0.4
0.2
0.0
-0.2
-0.4
-0.6
-0.8
-1.0
C Total cholesterol
Δ mmolL-1
1.5
1.0
0.5
0.0
-0.5
-1.0
-1.5
D HDL cholesterol
Δ mmolL-1
1.4
1.2
1.0
0.8
0.6
0.4
0.2
0.0
-0.2
-0.4
-0.6
E Systolic BP
Δ mm Hg
30
20
10
0
-10
-20
-30
-40
F Diastolic BP
Δ mm Hg
15
10
5
0
-5
-10
-15
Standard broccoli
HG broccoli
Peas
Standard broccoli
HG broccoli
Peas

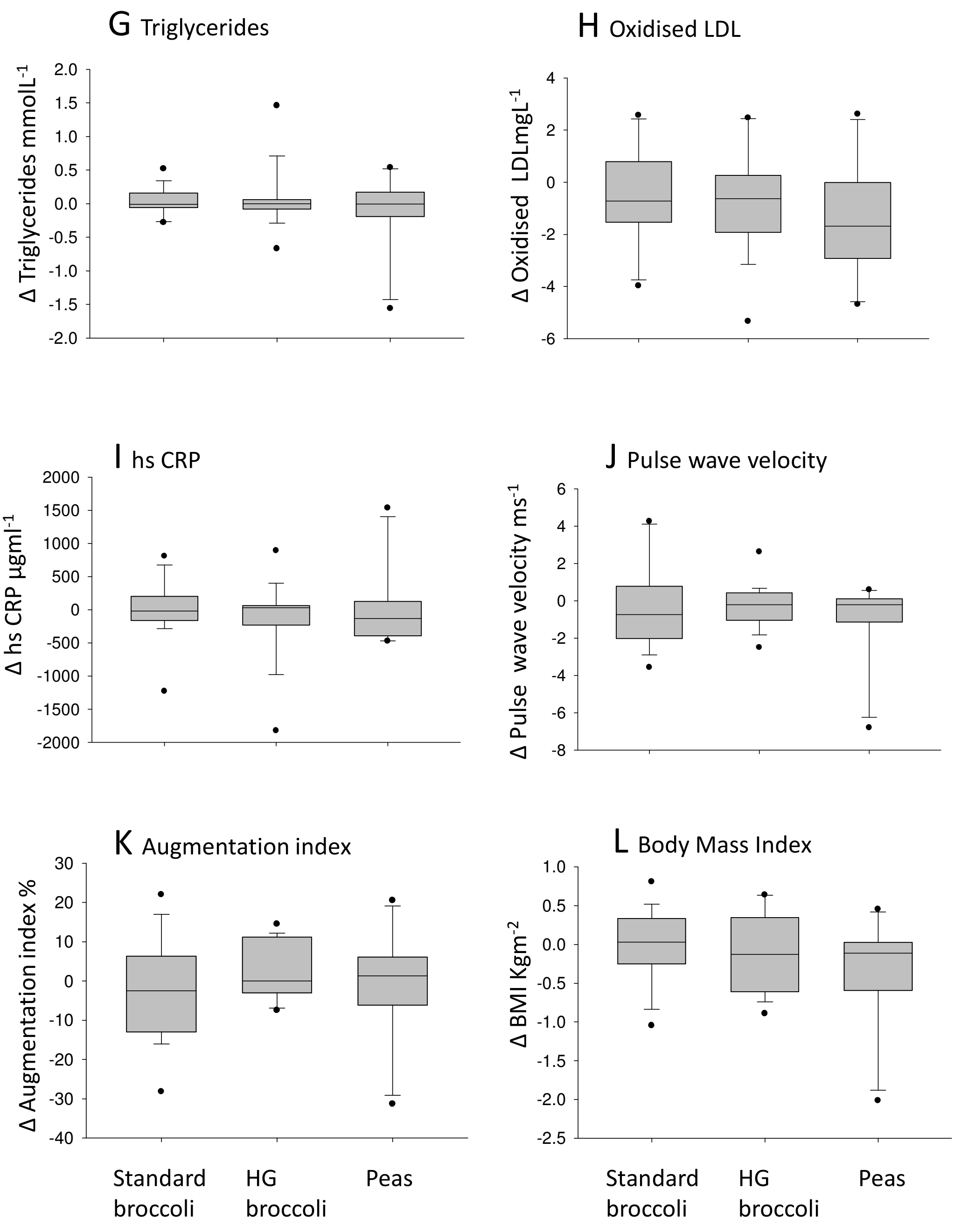

SUPPLEMENTAL FIGURE S2 Box plots showing changes in biomarkers associated with CVD risk following the 12 week. There are no significant differences between the changes observed amongst the three dietary arms

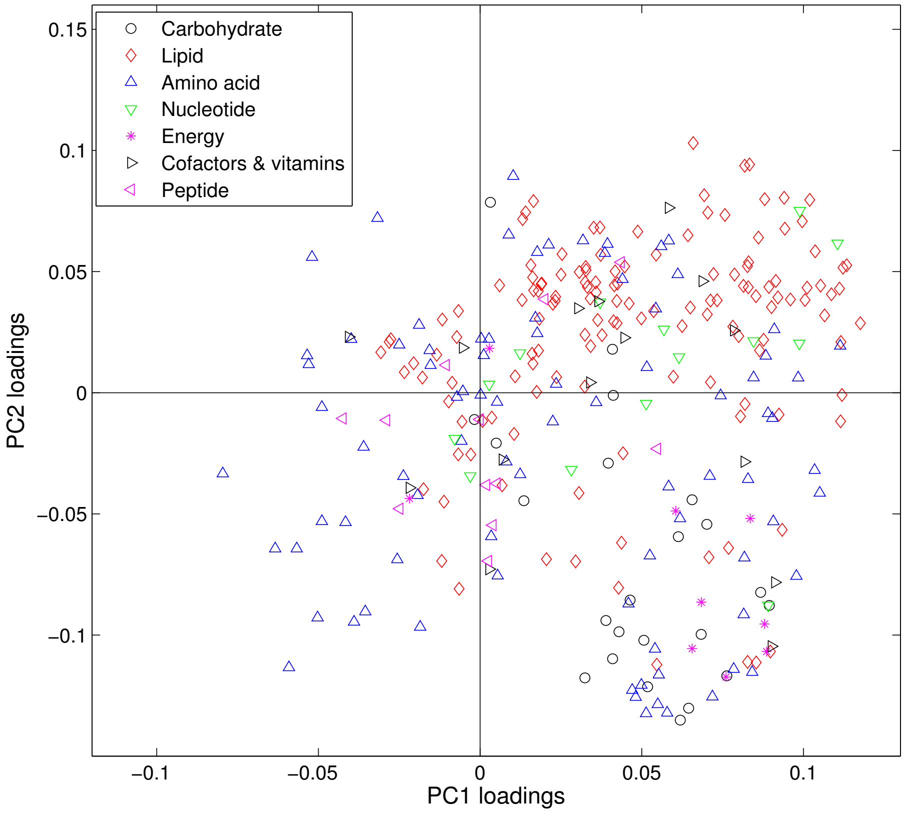

SUPPLEMENTAL FIGURE S3. PCA of metabolomics data, ratio post/pre-intervention. Loadings on 1st & 2nd axes showing the metabolites in terms of their super-pathways (N=48).

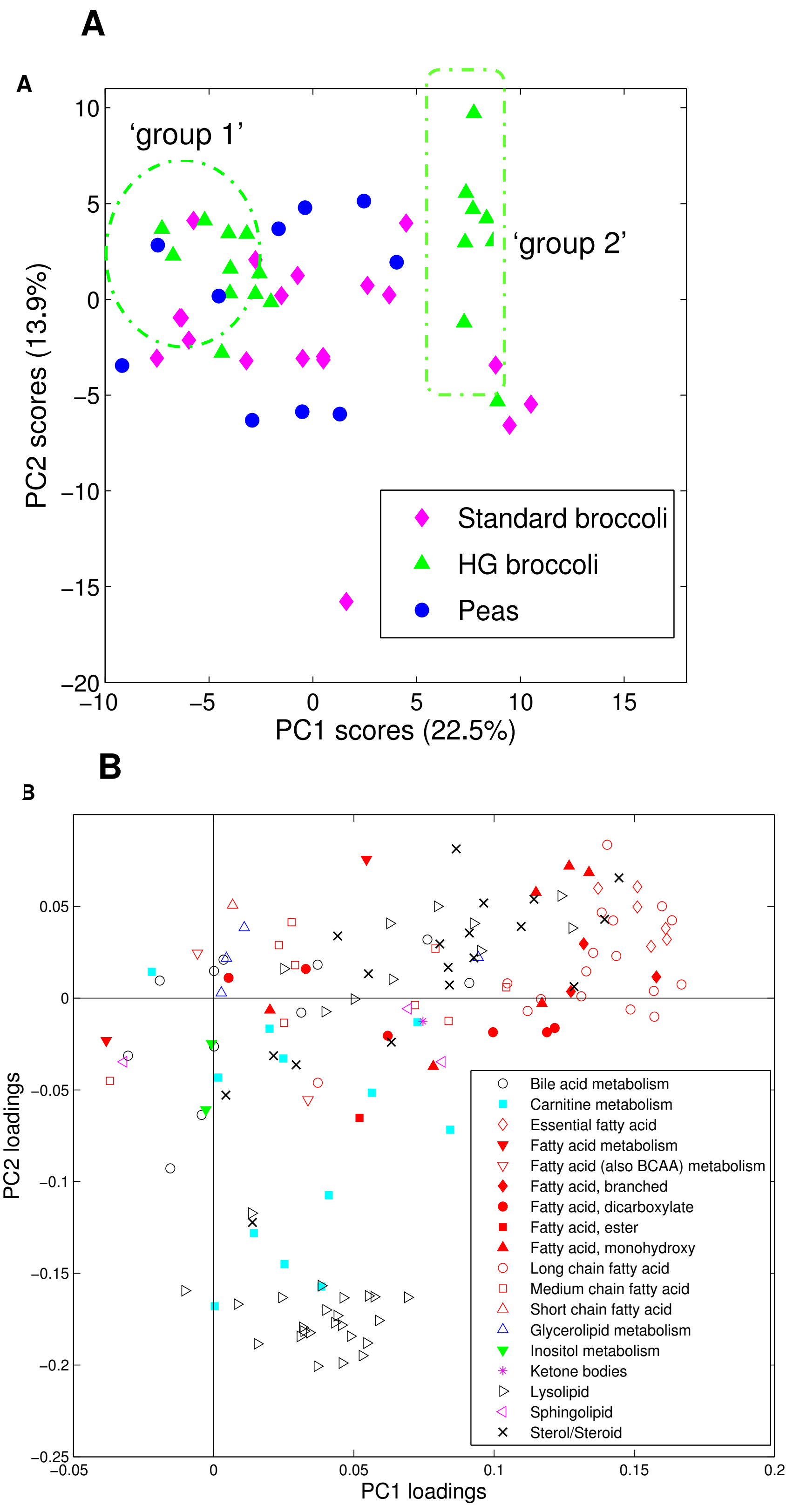

SUPPLEMENTAL FIGURE S4. PCA of lipid data, all volunteers, ratio post/pre-intervention. (A) Scores plot, and (B) loadings on 1st & 2nd axes showing the metabolites in terms of their sub-pathways (N=48).
