# Supplementary material for: A diet rich in high-glucoraphanin broccoli interacts with genotype to reduce discordance in plasma metabolite profiles by modulating mitochondrial function1
Source: Am J Clin Nutr. 2013 Aug 14;98(3):712–22. doi: 10.3945/ajcn.113.065235 (PMC3743733; doi:10.3945/ajcn.113.065235)
Supplement: Supplemental data [file 98.3.712_ajcn065235SupplementaryData2.pdf]

SUPPLEMENTAL TABLE S1

Dietary components significantly different between men and women. Results are of the mean values obtained from the two seven day diet dairies from 24 men and 24 women completed at baseline and after ten weeks.

	Male (n=24)	Female (n=24)	P
Energy kJ	9616 ± 2428	7902 ± 1845	0.008 [1]
Fat g	93.4 ± 32.59	73.7 ± 19.83	0.014
Protein g	87.5 ± 17.75	77.9 ± 13.91	0.042
Na g	3.2 ± 1.03	2.6 ± 0.74	0.024
Cl g	4.8 ± 1.48	3.9 ± 1.17	0.025
Alcohol g	14.6 ± 10.99	7.7 ± 10.26	0.029

[1] P values from T-tests

SUPPLEMENTAL TABLE S2

The p values that the differences between biomarkers of cardiovascular health occurred by chance

	Diet	Gender	PAPOLG	GSTM1	Diet x gender	Diet x PAPOLG	Diet X GSTM1	Gender X PAPOLG	Gender X GSTM1
Systolic blood pressure	0.72[1]	0.54	0.99	0.30	0.41	0.63	0.96	0.85	0.93
Diastolic blood pressure	0.92	0.72	0.89	0.63	0.49	0.98	0.68	0.96	0.77
Pulse wave velocity	0.79	**0.03**	0.23	**0.01**	0.55	**0.01**	**0.05**	0.52	0.04
Augmentation index	0.43	0.07	0.86	0.68	0.63	0.52	0.24	0.62	0.45
Body mass index	0.29	0.79	0.63	**0.05**	0.33	0.15	0.07	0.46	0.21
Weight	0.36	0.89	0.61	0.07	0.35	0.15	0.05	0.46	0.26
Total Cholesterol	0.38	0.53	0.29	0.86	0.27	0.41	0.57	0.22	0.64
HDL cholesterol	0.91	0.60	0.09	0.87	0.29	0.24	0.99	0.29	0.47
LDL cholesterol	0.29	0.39	0.29	0.79	0.21	0.71	0.63	0.51	0.95
Trigylcerides	0.72	0.14	0.36	0.79	0.70	0.98	0.32	0.90	0.91
CRP	0.38	0.68	0.16	0.37	0.21	0.91	0.92	0.61	0.43
Oxidised LDL	0.60	0.72	0.91	0.83	0.34	0.95	0.75	0.21	0.68
CVD 10 year risk	0.29	0.68	0.60	0.36	0.80	0.91	0.92	0.60	0.43

[1] P values derived from ANCOVA of the post intervention values with the pre-intervention values as co-variates in the model.

SUPPLEMENTAL TABLE S3

Details of the three SNPs that differentiate the two phenotype groups within the HG broccoli intervention arm

SNP	Position in DNA sequence and base change in PAPOLG		Frequency of major allele in study population %	Frequency of major allele previously reported % [1]
rs7579240	-15544 G>T	Upstream	72.6	65.5
rs28459296	11074 A>G	Intron 3	72.6	66.4
rs11687951	19963 G>C	Intron 8	72.6	66.8

[1] data summarised from http://www.ncbi.nlm.nih.gov for each individual SNP

SUPPLEMENTAL TABLE S4

P values of effect of Gender, Diet, Genotype, genotype x diet, gender x genotype and genotype x diet (n=48)

Pathway	Biochemical	Gender	Diet	Gene	Gender x diet	Gender x gene	Diet x gene
Alanine_aspartate_metabolism	alanine	0.281[1]	0.458	0.761	0.545	0.030	0.111
Alanine_aspartate_metabolism	asparagine	0.266	0.669	0.639	0.586	0.521	0.410
Alanine_aspartate_metabolism	aspartate	0.973	0.909	0.214	0.335	0.484	0.757
Alanine_aspartate_metabolism	N-acetylalanine	0.333	0.522	0.538	0.224	0.517	0.133
Alanine_aspartate_metabolism	N-acetyl-beta-alanine	0.142	0.339	0.472	0.066	0.875	0.043
Butanoate_metabolism	2-aminobutyrate	0.227	0.329	0.916	0.023	0.353	0.882
Creatine_metabolism	creatine	0.049	0.291	0.224	0.670	0.399	0.746
Creatine_metabolism	creatinine	0.025	0.659	0.477	0.328	0.189	0.819
Cysteine_methionine_SAM_taurine_metabolism	2-hydroxybutyrate (AHB)	0.712	0.874	0.048	0.538	0.437	0.388
Cysteine_methionine_SAM_taurine_metabolism	cysteine	0.689	0.727	0.088	0.509	0.965	0.395
Cysteine_methionine_SAM_taurine_metabolism	cystine	0.284	0.717	0.694	0.347	0.281	0.635
Cysteine_methionine_SAM_taurine_metabolism	methionine	0.632	0.084	0.012	0.955	0.618	0.387
Cysteine_methionine_SAM_taurine_metabolism	N-acetylmethionine	0.594	0.286	0.142	0.790	0.973	0.489
Cysteine_methionine_SAM_taurine_metabolism	N-formylmethionine	0.302	0.913	0.660	0.889	0.370	0.353
Cysteine_methionine_SAM_taurine_metabolism	S-methylcysteine	0.679	0.052	0.554	0.905	0.563	0.106
Glutamate_metabolism	glutamate	0.352	0.451	0.257	0.373	0.646	0.392
Glutamate_metabolism	glutamine	0.006	0.145	0.053	0.080	0.300	0.931
Glutamate_metabolism	pyroglutamine	0.488	0.535	0.393	0.127	0.312	0.330
Glutathione_metabolism	5-oxoproline	0.023	0.408	0.256	0.269	0.700	0.365

Glutathione_metabolism	glutathione, oxidized (GSSG)	0.460	0.881	0.917	0.969	0.353	0.939
Glycine_serine_threonine_metabolism	beta-hydroxypyruvate	0.961	0.735	0.243	0.515	0.330	0.781
Glycine_serine_threonine_metabolism	betaine	0.533	0.792	0.104	0.473	0.177	0.275
Glycine_serine_threonine_metabolism	dimethylglycine	0.648	0.223	0.807	0.829	0.647	0.885
Glycine_serine_threonine_metabolism	glycine	0.538	0.701	0.700	0.363	0.186	0.055
Glycine_serine_threonine_metabolism	N-acetylglycine	0.451	0.203	0.470	0.087	0.396	0.081
Glycine_serine_threonine_metabolism	N-acetylthreonine	0.043	0.774	0.829	0.285	0.212	0.670
Glycine_serine_threonine_metabolism	sarcosine (N-Methylglycine)	0.356	0.362	0.950	0.833	0.343	0.272
Glycine_serine_threonine_metabolism	serine	0.136	0.663	0.963	0.283	0.694	0.611
Glycine_serine_threonine_metabolism	threonine	0.849	0.964	0.228	0.755	0.427	0.061
Guanidino_acetamido_metabolism	4-acetamidobutanoate	0.508	0.309	0.144	0.597	0.906	0.273
Histidine_metabolism	3-methylhistidine	0.125	0.157	0.782	0.736	0.711	0.279
Histidine_metabolism	histidine	0.161	0.625	0.060	0.775	0.764	0.222
Lysine_metabolism	glutaroyl carnitine	0.520	0.408	0.365	0.342	0.670	0.226
Lysine_metabolism	lysine	0.869	0.043	0.640	0.331	0.465	0.089
Lysine_metabolism	N6-acetyllysine	0.124	0.999	0.795	0.288	0.326	0.137
Lysine_metabolism	pipecolate	0.583	0.427	0.796	0.320	0.946	0.797
Phenylalanine_tyrosine_metabolism	3-(4-hydroxyphenyl)lactate	0.168	0.375	0.065	0.171	0.691	0.080
Phenylalanine_tyrosine_metabolism	3-hydroxyphenylacetate	0.342	0.526	0.631	0.428	0.054	0.135
Phenylalanine_tyrosine_metabolism	3-methoxytyrosine	0.980	0.147	0.254	0.194	0.291	0.369
Phenylalanine_tyrosine_metabolism	3-phenylpropionate (hydrocinnamate)	0.689	0.413	0.012	0.517	0.803	0.479
Phenylalanine_tyrosine_metabolism	4-hydroxyphenylacetate	0.031	0.309	0.508	0.719	0.808	0.669
Phenylalanine_tyrosine_metabolism	4-hydroxyphenylpyruvate	0.371	0.085	0.809	0.955	0.819	0.475
Phenylalanine_tyrosine_metabolism	o-cresol sulfate	0.678	0.287	0.781	0.381	0.344	0.992
Phenylalanine_tyrosine_metabolism	p-cresol sulfate	0.358	0.188	0.273	0.674	0.973	0.541
Phenylalanine_tyrosine_metabolism	phenol sulfate	0.702	0.869	0.738	0.478	0.257	0.551
	phenylacetate	0.817	0.599	0.290	0.307	0.975	0.624

Phenylalanine_tyrosine_metabolism

Phenylalanine_tyrosine_metabolism	phenylacetylglutamine	0.977	0.391	0.578	0.758	0.926	0.730
Phenylalanine_tyrosine_metabolism	phenylalanine	0.187	0.521	0.176	0.953	0.276	0.521
Phenylalanine_tyrosine_metabolism	phenyllactate (PLA)	0.003	0.942	0.144	0.391	0.310	0.296
Phenylalanine_tyrosine_metabolism	tyrosine	0.316	0.993	0.005	0.646	0.937	0.467
Tryptophan_metabolism	3-indoxyl sulfate	0.329	0.677	0.451	0.675	0.541	0.376
Tryptophan_metabolism	C-glycosyltryptophan	0.449	0.037	0.898	0.630	0.402	0.945
Tryptophan_metabolism	indoleacetate	0.226	0.295	0.197	0.113	0.830	0.397
Tryptophan_metabolism	indolelactate	0.534	0.543	0.887	0.989	0.870	0.887
Tryptophan_metabolism	indolepropionate	0.571	0.416	0.467	0.301	0.173	0.620
Tryptophan_metabolism	kynurenine	0.957	0.725	0.525	0.878	0.684	0.658
Tryptophan_metabolism	serotonin (5HT)	0.851	0.945	0.119	0.080	0.477	0.907
Tryptophan_metabolism	tryptophan	0.695	0.515	0.273	0.981	0.638	0.863
Tryptophan_metabolism	tryptophan betaine	0.305	0.642	0.743	0.131	0.649	0.591
Urea_cycle_arginine_proline_metabolism	arginine	0.556	0.089	0.077	0.078	0.328	0.158
Urea_cycle_arginine_proline_metabolism	citrulline	0.276	0.364	0.355	0.245	0.664	0.534
Urea_cycle_arginine_proline_metabolism	N-acetylornithine	0.754	0.572	0.708	0.891	0.930	0.677
Urea_cycle_arginine_proline_metabolism	N-methyl proline	0.850	0.664	0.912	0.139	0.368	0.918
Urea_cycle_arginine_proline_metabolism	ornithine	0.823	0.870	0.246	0.922	0.571	0.534
Urea_cycle_arginine_proline_metabolism	proline	0.821	0.936	0.103	0.889	0.295	0.622
Urea_cycle_arginine_proline_metabolism	symmetric dimethylarginine (SDMA)	0.148	0.943	0.218	0.676	0.927	0.140
Urea_cycle_arginine_proline_metabolism	trans-4-hydroxyproline	0.448	0.989	0.107	0.464	0.373	0.892
Urea_cycle_arginine_proline_metabolism	urea	0.586	0.834	0.924	0.645	0.360	0.070
Valine_leucine_isoleucine_metabolism	2-hydroxy-3-methylvalerate	0.144	0.499	0.142	0.843	0.273	0.071
Valine_leucine_isoleucine_metabolism	2-hydroxyisobutyrate	0.724	0.045	0.494	0.995	0.682	0.694
Valine_leucine_isoleucine_metabolism	2-methylbutyroylcarnitine	0.925	0.774	0.958	0.809	0.852	0.921
	3-hydroxyisobutyrate	0.703	0.540	0.532	0.380	0.595	0.511

Valine_leucine_isoleucine_metabolism							
Valine_leucine_isoleucine_metabolism	3-methyl-2-oxobutyrate	0.063	0.111	0.005	0.720	0.640	0.048
Valine_leucine_isoleucine_metabolism	3-methyl-2-oxovalerate	0.025	0.306	0.014	0.844	0.152	0.042
Valine_leucine_isoleucine_metabolism	4-methyl-2-oxopentanoate	0.002	0.196	0.007	0.356	0.449	0.112
Valine_leucine_isoleucine_metabolism	alpha-hydroxyisocaproate	0.012	0.639	0.013	0.437	0.712	0.007
Valine_leucine_isoleucine_metabolism	alpha-hydroxyisovalerate	0.349	0.408	0.232	0.362	0.029	0.321
Valine_leucine_isoleucine_metabolism	beta-hydroxyisovalerate	0.052	0.350	0.203	0.896	0.917	0.974
Valine_leucine_isoleucine_metabolism	hydroxyisovaleroyl carnitine	0.608	0.218	0.684	0.086	0.532	0.359
Valine_leucine_isoleucine_metabolism	isobutyrylcarnitine	0.370	0.993	0.965	0.641	0.965	0.243
Valine_leucine_isoleucine_metabolism	isoleucine	0.781	0.434	0.280	0.331	0.181	0.762
Valine_leucine_isoleucine_metabolism	isovalerylcarnitine	0.755	0.911	0.784	0.177	0.869	0.395
Valine_leucine_isoleucine_metabolism	leucine	0.121	0.999	0.470	0.941	0.868	0.829
Valine_leucine_isoleucine_metabolism	tiglyl carnitine	0.269	0.401	0.996	0.063	0.585	0.947
Valine_leucine_isoleucine_metabolism	valine	0.045	0.838	0.792	0.484	0.216	0.852
Advanced_glycation_endproduct	erythrulose	0.011	0.677	0.003	0.909	0.530	0.359
Aminosugars_metabolism	erythronate	0.092	0.870	0.325	0.755	0.444	0.779
Aminosugars_metabolism	fucose	0.281	0.182	0.921	0.244	0.755	0.768
Fructose_mannose_galactose_starch_sucrose_metabolism	fructose	0.039	0.967	0.389	0.046	0.919	0.769
Fructose_mannose_galactose_starch_sucrose_metabolism	maltose	0.005	0.660	0.049	0.231	0.166	0.535
Fructose_mannose_galactose_starch_sucrose_metabolism	maltotetraose	0.022	0.362	0.463	0.127	0.360	0.713
Fructose_mannose_galactose_starch_sucrose_metabolism	maltotriose	0.008	0.858	0.090	0.262	0.344	0.755
Fructose_mannose_galactose_starch_sucrose_metabolism	mannose	0.038	0.475	0.270	0.589	0.778	0.781
Fructose_mannose_galactose_starch_sucrose_metabolism	methyl-beta-glucopyranoside	0.213	0.438	0.732	0.002	0.594	0.106
Glycolysis_gluconeogenesis_pyruvate_metabolism	1,5-anhydroglucitol (1,5-AG)	0.889	0.904	0.206	0.920	0.991	0.765
Glycolysis_gluconeogenesis_pyruvate_metabolism	1,6-anhydroglucose	0.044	0.724	0.674	0.313	0.177	0.648
Glycolysis_gluconeogenesis_pyruvate_metabolism	3-phosphoglycerate	0.326	0.888	0.010	0.866	0.474	0.208
	glucose	0.030	0.371	0.270	0.130	0.719	0.331

Glycolysis_gluconeogenesis_pyruvate_metabolism							
Glycolysis_gluconeogenesis_pyruvate_metabolism	glucuronate	0.110	0.471	0.169	0.322	0.397	0.607
Glycolysis_gluconeogenesis_pyruvate_metabolism	glycerate	0.329	0.653	0.049	0.295	0.918	0.955
Glycolysis_gluconeogenesis_pyruvate_metabolism	lactate	0.943	0.990	0.267	0.176	0.053	0.145
Glycolysis_gluconeogenesis_pyruvate_metabolism	pyruvate	0.371	0.769	0.523	0.440	0.547	0.346
Glyoxylate_dicarboxylate_metabolism	oxalate (ethanedioate)	0.879	0.251	0.065	0.576	0.204	0.348
Nucleotide_sugars_pentose_metabolism	arabinose	0.295	0.055	0.571	0.457	0.892	0.975
Nucleotide_sugars_pentose_metabolism	arabitol	0.031	0.580	0.988	0.327	0.078	0.087
Nucleotide_sugars_pentose_metabolism	ribitol	0.295	0.587	0.265	0.335	0.652	0.411
Nucleotide_sugars_pentose_metabolism	threitol	0.627	0.478	0.880	0.809	0.353	0.585
Nucleotide_sugars_pentose_metabolism	xylose	0.490	0.410	0.950	0.723	0.745	0.409
Ascorbate_aldarate_metabolism	threonate	0.389	0.942	0.021	0.284	0.735	0.739
Hemoglobin_porphyrin_metabolism	bilirubin (E,E)	0.058	0.828	0.105	0.609	0.135	0.845
Hemoglobin_porphyrin_metabolism	bilirubin (E,Z or Z,E)	0.329	0.888	0.674	0.407	0.244	0.086
Hemoglobin_porphyrin_metabolism	bilirubin (Z,Z)	0.020	0.643	0.109	0.578	0.022	0.997
Hemoglobin_porphyrin_metabolism	biliverdin	0.025	0.781	0.003	0.057	0.954	0.605
Hemoglobin_porphyrin_metabolism	heme	0.007	0.277	0.294	0.556	0.873	0.474
Hemoglobin_porphyrin_metabolism	L-urobilin	0.427	0.232	0.190	0.298	0.879	0.917
Nicotinate_nicotinamide_metabolism	adenosine 5'diphosphoribose	0.063	0.382	0.023	0.382	0.198	0.078
Nicotinate_nicotinamide_metabolism	nicotinamide	0.715	0.972	0.074	0.389	0.191	0.656
Nicotinate_nicotinamide_metabolism	trigonelline (N'-methylnicotinate)	0.683	0.574	0.338	0.819	0.826	0.549
Pantothenate_CoA_metabolism	pantothenate	0.887	0.691	0.832	0.907	0.586	0.304
Riboflavin_metabolism	flavin adenine dinucleotide (FAD)	0.604	0.077	0.001	0.898	0.843	0.052
Tocopherol_metabolism	alpha-tocopherol	0.180	0.005	0.137	0.925	0.357	0.223
Tocopherol_metabolism	gamma-CEHC	0.427	0.656	0.545	0.987	0.803	0.553
Vitamin_B6_metabolism	pyridoxate	0.192	0.614	0.241	0.340	0.473	0.879
	alpha-ketoglutarate	0.121	0.084	0.237	0.271	0.081	0.242

Krebs_cycle

Krebs_cycle	citrate	0.982	0.863	0.533	0.034	0.532	0.872
Krebs_cycle	fumarate	0.946	0.939	0.798	0.930	0.418	0.880
Krebs_cycle	malate	0.155	0.433	0.289	0.310	0.123	0.865
Krebs_cycle	succinate	0.571	0.714	0.686	0.443	0.105	0.018
Krebs_cycle	succinylcarnitine	0.375	0.934	0.971	0.494	0.389	0.245
Oxidative_phosphorylation	acetylphosphate	0.562	0.602	0.457	0.213	0.057	0.322
Oxidative_phosphorylation	phosphate	0.026	0.278	0.008	0.596	0.522	0.227
Oxidative_phosphorylation	pyrophosphate (PPi)	0.718	0.950	0.481	0.955	0.274	0.486
Bile_acid_metabolism	chenodeoxycholate	0.078	0.810	0.368	0.206	0.714	0.658
Bile_acid_metabolism	cholate	0.227	0.436	0.824	0.076	0.897	0.635
Bile_acid_metabolism	deoxycholate	0.235	0.737	0.144	0.886	0.038	0.516
Bile_acid_metabolism	glycochenodeoxycholate	0.831	0.539	0.988	0.546	0.777	0.138
Bile_acid_metabolism	glycocholate	0.619	0.174	0.429	0.373	0.329	0.605
Bile_acid_metabolism	glycocholenate sulfate	0.449	0.076	0.031	0.915	0.272	0.140
Bile_acid_metabolism	glycodeoxycholate	0.669	0.844	0.644	0.700	0.986	0.498
Bile_acid_metabolism	glycolithocholate sulfate	0.134	0.029	0.975	0.054	0.740	0.957
Bile_acid_metabolism	glycoursodeoxycholate	0.995	0.495	0.729	0.327	0.741	0.704
Bile_acid_metabolism	taurocholenate sulfate	0.208	0.390	0.301	0.048	0.528	0.174
Bile_acid_metabolism	taurolithocholate 3-sulfate	0.280	0.526	0.756	0.246	0.037	0.223
Carnitine_metabolism	3-dehydrocarnitine	0.063	0.455	0.473	0.347	0.202	0.237
Carnitine_metabolism	acetylcarnitine	0.815	0.827	0.268	0.917	0.604	0.720
Carnitine_metabolism	carnitine	0.579	0.309	0.514	0.361	0.787	0.537
Carnitine_metabolism	cis-4-decenoyl carnitine	0.615	0.343	0.230	0.800	0.179	0.814
Carnitine_metabolism	decanoylcarnitine	0.205	0.155	0.832	0.642	0.935	0.861
Carnitine_metabolism	deoxycarnitine	0.107	0.523	0.716	0.595	0.398	0.542
Carnitine_metabolism	hexanoylcarnitine	0.500	0.252	0.696	0.511	0.204	0.125

Carnitine_metabolism	laurylcarnitine	0.096	0.478	0.894	0.673	0.979	0.811
Carnitine_metabolism	octanoylcarnitine	0.247	0.393	0.865	0.583	0.496	0.506
Carnitine_metabolism	oleoylcarnitine	0.112	0.280	0.635	0.966	0.829	0.659
Carnitine_metabolism	palmitoylcarnitine	0.068	0.792	0.999	0.474	0.942	0.537
Carnitine_metabolism	stearoylcarnitine	0.015	0.854	0.783	0.276	0.791	0.748
Essential_fatty_acid	dihomo-linolenate (20:3n3 or n6)	0.896	0.387	0.004	0.392	0.425	0.013
Essential_fatty_acid	docosahexaenoate (DHA; 22:6n3)	0.842	0.240	0.005	0.228	0.760	0.003
Essential_fatty_acid	docosapentaenoate (n3 DPA; 22:5n3)	0.563	0.126	0.016	0.193	0.667	0.003
Essential_fatty_acid	eicosapentaenoate (EPA; 20:5n3)	0.603	0.116	0.051	0.232	0.969	0.003
Essential_fatty_acid	linoleate (18:2n6)	0.648	0.232	0.095	0.163	0.767	0.035
Essential_fatty_acid	linolenate [alpha or gamma; (18:3n3 or 6)]	0.894	0.230	0.143	0.385	0.892	0.010
Fatty_acid_branched	13-methylmyristic acid	0.382	0.030	0.187	0.550	0.285	0.072
Fatty_acid_branched	15-methylpalmitate (isobar with 2-methylpalmitate)	0.472	0.051	0.007	0.225	0.312	0.053
Fatty_acid_branched	17-methylstearate	0.923	0.071	0.026	0.428	0.149	0.348
Fatty_acid_dicarboxylate	3-carboxy-4-methyl-5-propyl-2-furanpropanoate (CMPF)	0.456	0.852	0.891	0.593	0.674	0.646
Fatty_acid_dicarboxylate	azelate (nonanedioate)	0.231	0.064	0.325	0.296	0.007	0.675
Fatty_acid_dicarboxylate	dodecanedioate	0.308	0.113	0.157	0.864	0.915	0.806
Fatty_acid_dicarboxylate	hexadecanedioate	0.087	0.587	0.860	0.514	0.716	0.056
Fatty_acid_dicarboxylate	octadecanedioate	0.206	0.963	0.359	0.979	0.476	0.346
Fatty_acid_dicarboxylate	tetradecanedioate	0.176	0.479	0.321	0.708	0.909	0.055
Fatty_acid_ester	n-Butyl Oleate	0.691	0.409	0.062	0.298	0.071	0.342
Fatty_acid_metabolism	butyrylcarnitine	0.850	0.428	0.174	0.060	0.550	0.551
Fatty_acid_metabolism	isovalerate	0.106	0.265	0.006	0.011	0.113	0.175
Fatty_acid_metabolism	propionylcarnitine	0.421	0.527	0.127	0.729	0.677	0.472
Fatty_acid_metabolism	valerylcarnitine	0.731	0.221	0.018	0.142	0.081	0.439
Fatty_acid_monohydroxy	13-HODE + 9-HODE	0.789	0.560	0.383	0.238	0.978	0.027
	2-hydroxyoctanoate	0.898	0.964	0.773	0.178	0.629	0.391

Fatty_acid_monohydroxy							
Fatty_acid_monohydroxy	2-hydroxypalmitate	0.536	0.959	0.025	0.116	0.610	0.013
Fatty_acid_monohydroxy	2-hydroxystearate	0.378	0.960	0.070	0.288	0.946	0.018
Fatty_acid_monohydroxy	3-hydroxydecanoate	0.076	0.824	0.423	0.805	0.947	0.093
Fatty_acid_monohydroxy	3-hydroxyoctanoate	0.103	0.335	0.896	0.945	0.488	0.858
Glycerolipid_metabolism	choline	0.885	0.906	0.189	0.283	0.721	0.806
Glycerolipid_metabolism	glycerol 3-phosphate (G3P)	0.020	0.027	0.410	0.289	0.413	0.024
Glycerolipid_metabolism	glycerophosphorylcholine (GPC)	0.086	0.878	0.462	0.655	0.578	0.886
Glycerolipid_metabolism	phosphoethanolamine	0.715	0.826	0.397	0.886	0.283	0.251
Glycerolipid_metabolism	trimethylamine N-oxide	0.257	0.105	0.347	0.148	0.030	0.985
Inositol_metabolism	chiro-inositol	0.552	0.404	0.394	0.151	0.421	0.414
Inositol_metabolism	myo-inositol	0.381	0.632	0.338	0.695	0.641	0.479
Ketone_bodies	3-hydroxybutyrate (BHBA)	0.542	0.302	0.631	0.404	0.482	0.888
Long_chain_fatty_acid	10-heptadecenoate (17:1n7)	0.800	0.172	0.110	0.208	0.223	0.020
Long_chain_fatty_acid	10-nonadecenoate (19:1n9)	0.304	0.170	0.010	0.408	0.138	0.017
Long_chain_fatty_acid	adrenate (22:4n6)	0.609	0.126	0.014	0.472	0.545	0.117
Long_chain_fatty_acid	arachidonate (20:4n6)	0.245	0.442	0.029	0.323	0.731	0.011
Long_chain_fatty_acid	cis-vaccenate (18:1n7)	0.572	0.466	0.700	0.691	0.750	0.029
Long_chain_fatty_acid	dihomo-linoleate (20:2n6)	0.491	0.057	0.005	0.294	0.830	0.021
Long_chain_fatty_acid	eicosenoate (20:1n9 or 11)	0.411	0.244	0.077	0.275	0.756	0.030
Long_chain_fatty_acid	margarate (17:0)	0.457	0.030	0.064	0.186	0.144	0.009
Long_chain_fatty_acid	myristate (14:0)	0.820	0.277	0.084	0.265	0.491	0.013
Long_chain_fatty_acid	myristoleate (14:1n5)	0.112	0.908	0.194	0.591	0.138	0.070
Long_chain_fatty_acid	nonadecanoate (19:0)	0.656	0.077	0.016	0.514	0.170	0.088
Long_chain_fatty_acid	oleate (18:1n9)	0.548	0.408	0.644	0.361	0.392	0.184
Long_chain_fatty_acid	palmitate (16:0)	0.551	0.183	0.044	0.188	0.726	0.018
	palmitoleate (16:1n7)	0.246	0.463	0.127	0.143	0.549	0.045

Long_chain_fatty_acid							
Long_chain_fatty_acid	pentadecanoate (15:0)	0.518	0.266	0.050	0.126	0.506	0.005
Long_chain_fatty_acid	stearate (18:0)	0.623	0.187	0.062	0.231	0.757	0.054
Long_chain_fatty_acid	stearidonate (18:4n3)	0.017	0.625	0.676	0.070	0.008	0.285
Lysolipid	1-arachidonoylglycerophosphocholine	0.036	0.433	0.096	0.161	0.799	0.687
Lysolipid	1-arachidonoylglycerophosphoethanolamine	0.330	0.648	0.001	0.084	0.286	0.038
Lysolipid	1-arachidonoylglycerophosphoinositol	0.650	0.429	0.037	0.397	0.092	0.006
Lysolipid	1-docosahexaenoylglycerophosphocholine	0.435	0.805	0.251	0.167	0.316	0.648
Lysolipid	1-docosapentaenoylglycerophosphocholine	0.035	0.488	0.384	0.052	0.501	0.846
Lysolipid	1-eicosadienoylglycerophosphocholine	0.220	0.304	0.037	0.250	0.075	0.601
Lysolipid	1-eicosatrienoylglycerophosphocholine	0.033	0.130	0.405	0.255	0.914	0.361
Lysolipid	1-heptadecanoylglycerophosphocholine	0.426	0.939	0.035	0.086	0.357	0.629
Lysolipid	1-linoleoylglycerophosphocholine	0.045	0.723	0.212	0.564	0.641	0.670
Lysolipid	1-linoleoylglycerophosphoethanolamine	0.184	0.859	0.282	0.848	0.789	0.661
Lysolipid	1-myristoylglycerophosphocholine	0.956	0.325	0.091	0.427	0.258	0.308
Lysolipid	1-oleoylglycerophosphocholine	0.075	0.326	0.120	0.330	0.143	0.441
Lysolipid	1-oleoylglycerophosphoethanolamine	0.775	0.226	0.125	0.771	0.235	0.007
Lysolipid	1-palmitoleoylglycerophosphocholine	0.340	0.381	0.111	0.528	0.392	0.586
Lysolipid	1-palmitoylglycerophosphocholine	0.078	0.708	0.078	0.179	0.193	0.447
Lysolipid	1-palmitoylglycerophosphoethanolamine	0.185	0.252	0.025	0.731	0.831	0.208
Lysolipid	1-palmitoylplasmenylethanolamine	0.861	0.778	0.005	0.366	0.588	0.133
Lysolipid	1-pentadecanoylglycerophosphocholine	0.197	0.511	0.146	0.377	0.225	0.684
Lysolipid	1-stearoylglycerophosphocholine	0.212	0.733	0.074	0.267	0.268	0.236
Lysolipid	1-stearoylglycerophosphoethanolamine	0.525	0.852	0.590	0.352	0.164	0.565
Lysolipid	1-stearoylglycerophosphoinositol	0.702	0.576	0.026	0.500	0.829	0.027
Lysolipid	2-arachidonoylglycerophosphocholine	0.030	0.074	0.051	0.137	0.139	0.459
	2-arachidonoylglycerophosphoethanolamine	0.084	0.651	0.087	0.092	0.371	0.408

Lysolipid							
Lysolipid	2-docosahexaenoylglycerophosphocholine	0.887	0.286	0.528	0.212	0.081	0.263
Lysolipid	2-docosahexaenoylglycerophosphoethanolamine	0.794	0.612	0.358	0.354	0.242	0.110
Lysolipid	2-linoleoylglycerophosphocholine	0.085	0.496	0.337	0.519	0.371	0.322
Lysolipid	2-linoleoylglycerophosphoethanolamine	0.635	0.560	0.014	0.144	0.371	0.252
Lysolipid	2-myristoylglycerophosphocholine	0.730	0.419	0.295	0.239	0.705	0.471
Lysolipid	2-oleoylglycerophosphocholine	0.030	0.139	0.395	0.110	0.171	0.228
Lysolipid	2-oleoylglycerophosphoethanolamine	0.261	0.269	0.407	0.948	0.298	0.334
Lysolipid	2-palmitoleoylglycerophosphocholine	0.529	0.415	0.069	0.593	0.243	0.649
Lysolipid	2-palmitoylglycerophosphocholine	0.066	0.981	0.075	0.145	0.798	0.330
Lysolipid	2-palmitoylglycerophosphoethanolamine	0.020	0.691	0.008	0.918	0.432	0.284
Lysolipid	2-stearoylglycerophosphocholine	0.385	0.915	0.066	0.174	0.165	0.430
Medium_chain_fatty_acid	10-undecenoate (11:1n1)	0.148	0.690	0.781	0.769	0.984	0.080
Medium_chain_fatty_acid	5-dodecenoate (12:1n7)	0.231	0.223	0.855	0.874	0.617	0.009
Medium_chain_fatty_acid	caprate (10:0)	0.532	0.076	0.215	0.732	0.890	0.308
Medium_chain_fatty_acid	caproate (6:0)	0.214	0.987	0.047	0.656	0.759	0.641
Medium_chain_fatty_acid	caprylate (8:0)	0.628	0.519	0.441	0.584	0.630	0.799
Medium_chain_fatty_acid	heptanoate (7:0)	0.237	0.998	0.493	0.666	0.177	0.216
Medium_chain_fatty_acid	laurate (12:0)	0.533	0.775	0.353	0.887	0.733	0.231
Medium_chain_fatty_acid	pelargonate (9:0)	0.120	0.362	0.106	0.886	0.989	0.286
Medium_chain_fatty_acid	undecanoate (11:0)	0.262	0.940	0.435	0.421	0.207	0.507
Short_chain_fatty_acid	valerate	0.680	0.588	0.463	0.513	0.678	0.789
Sphingolipid	palmitoyl sphingomyelin	0.408	0.020	0.679	0.914	0.463	0.129
Sphingolipid	sphingosine	0.742	0.808	0.302	0.054	0.250	0.677
Sphingolipid	stearoyl sphingomyelin	0.299	0.216	0.709	0.565	0.836	0.383
Sterol_Steroid	21-hydroxypregnenolone disulfate	0.068	0.250	0.027	0.073	0.878	0.518
	4-androsten-3beta,17beta-diol disulfate 1	0.300	0.042	0.938	0.026	0.629	0.555

Sterol_Steroid							
Sterol_Steroid	4-androsten-3beta,17beta-diol disulfate 2	0.030	0.175	0.034	0.016	0.786	0.252
Sterol_Steroid	5alpha-androstan-3beta,17beta-diol disulfate	0.531	0.097	0.860	0.296	0.505	0.990
Sterol_Steroid	5alpha-pregnan-3beta,20alpha-diol disulfate	0.021	0.973	0.503	0.088	0.547	0.556
Sterol_Steroid	7-alpha-hydroxy-3-oxo-4-cholestenoate (7-Hoca)	0.171	0.091	0.719	0.180	0.919	0.002
Sterol_Steroid	andro steroid monosulfate 1	0.488	0.062	0.190	0.778	0.264	0.871
Sterol_Steroid	andro steroid monosulfate 2	0.001	0.359	0.062	0.054	0.519	0.433
Sterol_Steroid	androsterone sulfate	0.205	0.121	0.930	0.743	0.874	0.043
Sterol_Steroid	beta-sitosterol	0.757	0.215	0.157	0.251	0.451	0.315
Sterol_Steroid	cholesterol	0.098	0.248	0.305	0.666	0.523	0.262
Sterol_Steroid	cortisol	0.243	0.391	0.875	0.696	0.443	0.403
Sterol_Steroid	cortisone	0.822	0.942	0.276	0.508	0.196	0.312
Sterol_Steroid	dehydroisoandrosterone sulfate (DHEA-S)	0.005	0.042	0.012	0.003	0.819	0.001
Sterol_Steroid	epiandrosterone sulfate	0.340	0.092	0.420	0.432	0.764	0.008
Sterol_Steroid	lathosterol	0.135	0.668	0.597	0.085	0.032	0.842
Sterol_Steroid	pregn steroid monosulfate	0.039	0.249	0.278	0.044	0.773	0.010
Sterol_Steroid	pregnen-diol disulfate	0.217	0.352	0.433	0.063	0.675	0.829
Sterol_Steroid	pregnenolone sulfate	0.294	0.156	0.002	0.158	0.485	0.198
Sterol_Steroid	squalene	0.294	0.194	0.657	0.391	0.091	0.089
Purine_metabolism_adenine_containing	adenosine 5'-diphosphate (ADP)	0.371	0.450	0.218	0.905	0.523	0.074
Purine_metabolism_adenine_containing	adenosine 5'-monophosphate (AMP)	0.431	0.654	0.220	0.518	0.152	0.954
Purine_metabolism_adenine_containing	N1-methyladenosine	0.897	0.198	0.036	0.221	0.586	0.896
Purine_metabolism_guanine_containing	N1-methylguanosine	0.266	0.877	0.452	0.113	0.102	0.332
Purine_metabolism_guanine_containing	N2,N2-dimethylguanosine	0.474	0.162	0.326	0.810	0.231	0.147
Purine_metabolism_guanine_containing	N6-carbamoylthreonyladenosine	0.214	0.569	0.248	0.191	0.169	0.491
Purine_metabolism_hypoxanthine_inosine_containing	hypoxanthine	0.701	0.609	0.478	0.331	0.725	0.099
	xanthine	0.521	0.032	0.497	0.088	0.191	0.998

Purine_metabolism_hypoxanthine_inosine_containing							
Purine_metabolism_urate_metabolism	urate	0.006	0.387	0.040	0.270	0.528	0.119
Purine_pyrimidine_metabolism	methylphosphate	0.211	0.641	0.379	0.551	0.864	0.623
Pyrimidine_metabolism_cytidine_containing	N4-acetylcytidine	0.407	0.002	0.343	0.981	0.313	0.470
Pyrimidine_metabolism_uracil_containing	5-methyluridine (ribothymidine)	0.470	0.310	0.008	0.897	0.692	0.277
Pyrimidine_metabolism_uracil_containing	pseudouridine	0.072	0.812	0.057	0.064	0.708	0.018
Pyrimidine_metabolism_uracil_containing	uridine	0.858	0.987	0.071	0.901	0.932	0.023
Dipeptide	aspartylphenylalanine	0.731	0.791	0.325	0.730	0.862	0.112
Dipeptide	cyclo(leu-pro)	0.700	0.254	0.159	0.595	0.127	0.768
Dipeptide	phenylalanylphenylalanine	0.552	0.905	0.833	0.950	0.927	0.334
Dipeptide	pro-hydroxy-pro	0.667	0.534	0.104	0.333	0.863	0.663
gammaglutamyl	gamma-glutamylalanine	0.250	0.727	0.171	0.670	0.643	0.107
gammaglutamyl	gamma-glutamylglutamine	0.169	0.653	0.522	0.253	0.881	0.100
gammaglutamyl	gamma-glutamylisoleucine	0.419	0.496	0.602	0.347	0.519	0.576
gammaglutamyl	gamma-glutamylleucine	0.342	0.471	0.878	0.363	0.783	0.068
gammaglutamyl	gamma-glutamylmethionine	0.298	0.008	0.937	0.668	0.741	0.607
gammaglutamyl	gamma-glutamylphenylalanine	0.091	0.267	0.195	0.808	0.432	0.473
gammaglutamyl	gamma-glutamyltyrosine	0.543	0.392	0.084	0.443	0.886	0.207
gammaglutamyl	gamma-glutamylvaline	0.942	0.551	0.503	0.875	0.980	0.973
Polypeptide	bradykinin	0.323	0.459	0.226	0.452	0.242	0.387
Polypeptide	bradykinin, hydroxy-pro(3)	0.962	0.140	0.060	0.234	0.805	0.080
Benzoate_metabolism	2-hydroxyhippurate (salicylurate)	0.648	0.883	0.922	0.753	0.288	0.513
Benzoate_metabolism	3-hydroxyhippurate	0.796	0.904	0.567	0.332	0.186	0.505
Benzoate_metabolism	4-ethylphenylsulfate	0.603	0.113	0.237	0.922	0.726	0.382
Benzoate_metabolism	4-vinylphenol sulfate	0.835	0.771	0.581	0.952	0.174	0.287
Benzoate_metabolism	benzoate	0.000	0.836	0.430	0.311	0.058	0.239
	catechol sulfate	0.333	0.165	0.697	0.014	0.937	0.502

Benzoate_metabolism							
Benzoate_metabolism	hippurate	0.926	0.350	0.035	0.032	0.601	0.719
Benzoate_metabolism	methyl-4-hydroxybenzoate	0.044	0.789	0.588	0.949	0.610	0.565
Chemical	2-ethylhexanoate	0.097	0.545	0.569	0.966	0.086	0.805
Chemical	diisopropanolamine	0.933	0.865	0.933	0.601	0.081	0.158
Chemical	glycerol 2-phosphate	0.983	0.234	0.685	0.744	0.094	0.184
Chemical	glycolate (hydroxyacetate)	0.739	0.151	0.611	0.864	0.694	0.766
Chemical	iminodiacetate (IDA)	0.689	0.025	0.796	0.438	0.267	0.600
Drug	2-hydroxyacetaminophen sulfate	0.787	0.758	0.256	0.638	0.429	0.364
Drug	2-methoxyacetaminophen glucuronide	0.730	0.929	0.228	0.323	0.995	0.069
Drug	2-methoxyacetaminophen sulfate	0.602	0.642	0.198	0.347	0.915	0.098
Drug	3-(cystein-S-yl)acetaminophen	0.873	0.531	0.188	0.102	0.873	0.248
Drug	4-acetamidophenol	0.937	0.275	0.219	0.133	0.750	0.119
Drug	4-acetaminophen sulfate	0.821	0.730	0.218	0.668	0.406	0.358
Drug	codeine	0.616	0.714	0.152	0.038	0.222	0.332
Drug	desmethylnaproxen sulfate	0.614	0.311	0.483	0.030	0.458	0.091
Drug	ibuprofen	0.529	0.197	0.205	0.522	0.241	0.359
Drug	naproxen	0.966	0.251	0.751	0.515	0.915	0.387
Drug	p-acetamidophenylglucuronide	0.498	0.521	0.259	0.639	0.679	0.173
Drug	pivaloylcarnitine	0.457	0.777	0.359	0.332	0.743	0.260
Drug	pseudoephedrine	0.053	0.462	0.572	0.628	0.469	0.240
Drug	quinine	0.816	0.567	0.570	0.589	0.472	0.422
EDTA	EDTA	0.154	0.898	0.269	0.616	0.325	0.170
Food_component_Plant	cinnamoylglycine	0.271	0.871	0.070	0.419	0.969	0.149
Food_component_Plant	homostachydrine	0.732	0.046	0.975	0.463	0.436	0.078
Food_component_Plant	piperine	0.224	0.293	0.457	0.086	0.894	0.169
	quinate	0.780	0.102	0.692	0.423	0.696	0.535

Food_component_Plant							
Food_component_Plant	saccharin	0.035	0.177	0.165	0.095	0.173	0.175
Food_component_Plant	stachydrine	0.906	0.957	0.958	0.435	0.859	0.144
Food_component_Plant	thymol sulfate	0.281	0.068	0.056	0.749	0.111	0.588
Sugar_sugar_substitute_starch	erythritol	0.553	0.858	0.603	0.316	0.132	0.777
Tobacco_metabolite	cotinine	0.161	0.906	0.983	0.549	0.196	0.930
Xanthine_metabolism	1,7-dimethylurate	0.258	0.662	0.835	0.118	0.121	0.461
Xanthine_metabolism	1-methylurate	0.072	0.613	0.061	0.035	0.541	0.987
Xanthine_metabolism	1-methylxanthine	0.020	0.021	0.120	0.009	0.531	0.069
Xanthine_metabolism	3-methylxanthine	0.572	0.117	0.066	0.880	0.130	0.044
Xanthine_metabolism	7-methylxanthine	0.235	0.514	0.752	0.838	0.635	0.076
Xanthine_metabolism	caffeine	0.676	0.679	0.247	0.547	0.139	0.893
Xanthine_metabolism	paraxanthine	0.386	0.870	0.653	0.167	0.188	0.946
Xanthine_metabolism	theobromine	0.244	0.217	0.834	0.893	0.113	0.106
Xanthine_metabolism	theophylline	0.262	0.927	0.464	0.167	0.623	0.580

[1] ANCOVA was used to calculate P values for the likelihood of significant differences between individual metabolites. For each metabolite, the post-intervention value of the log2 ion intensity was used as the response variable and the pre-intervention value was used as a covariate. Gender, diet and genotype were factor variables and their main effects and interaction were estimated in the model. Yellow denotes P value <0.05.

SUPPLEMENTAL TABLE S5

P values of differences in metabolites for genotypes 1 and 2 before and after the study in the HG broccoli arm (n=19)

SUPER PATHWAY	SUB PATHWAY	BIOCHEMICAL	Pre 1 v 2	Post 1 v2	1 Pre post	2 Pre post
Amino acid	Glycine, serine and threonine metabolism	glycine	0.943[1]	0.712	0.818	0.911
Amino acid	Glycine, serine and threonine metabolism	sarcosine (N-Methylglycine)	0.712	0.215	0.072	0.548
Amino acid	Glycine, serine and threonine metabolism	dimethylglycine	0.421	0.495	0.434	0.994
Amino acid	Glycine, serine and threonine metabolism	N-acetylglycine	0.041 [1]	0.454	0.168	0.032 [1]
Amino acid	Glycine, serine and threonine metabolism	beta-hydroxypyruvate	0.488	0.137	0.896	0.194
Amino acid	Glycine, serine and threonine metabolism	serine	0.625	0.948	0.527	0.838
Amino acid	Glycine, serine and threonine metabolism	threonine	0.045 [1]	0.055	0.275	0.001 [1]
Amino acid	Glycine, serine and threonine metabolism	N-acetylthreonine	0.058	0.797	0.468	0.082
Amino acid	Glycine, serine and threonine metabolism	betaine	0.825	0.151	0.084	0.111
Amino acid	Alanine and aspartate metabolism	aspartate	0.151	0.786	0.014	0.669
Amino acid	Alanine and aspartate metabolism	asparagine	0.573	0.834	0.642	0.864
Amino acid	Alanine and aspartate metabolism	N-acetyl-beta-alanine	0.851	0.061	0.634	0.191
Amino acid	Alanine and aspartate metabolism	alanine	0.456	0.907	0.335	0.978
Amino acid	Alanine and aspartate metabolism	N-acetylalanine	0.022	0.166	0.291	0.037
Amino acid	Glutamate metabolism	glutamate	0.193	0.918	0.298	0.753
Amino acid	Glutamate metabolism	glutamine	0.054	0.423	0.749	0.008
Amino acid	Glutamate metabolism	pyroglutamine*	0.521	0.468	0.634	0.687
Amino acid	Histidine metabolism	histidine	0.715	0.020	0.695	0.006
Amino acid	Histidine metabolism	3-methylhistidine	0.059	0.007	0.846	0.744
Amino acid	Lysine metabolism	lysine	0.681	0.154	0.549	0.032
Amino acid	Lysine metabolism	pipecolate	0.414	0.508	0.785	0.869
Amino acid	Lysine metabolism	N6-acetyllysine	0.267	0.463	0.537	0.998

Amino acid	Lysine metabolism	glutaroyl carnitine	0.808	0.680	0.256	0.658
Amino acid	Phenylalanine & tyrosine metabolism	phenyllactate (PLA)	0.989	0.160	0.239	0.314
Amino acid	Phenylalanine & tyrosine metabolism	phenylalanine	0.048	0.482	0.872	0.088
Amino acid	Phenylalanine & tyrosine metabolism	phenylacetate	0.023	0.565	0.963	0.066
Amino acid	Phenylalanine & tyrosine metabolism	p-cresol sulfate	0.256	0.510	0.291	0.275
Amino acid	Phenylalanine & tyrosine metabolism	o-cresol sulfate	0.089	0.287	0.341	0.852
Amino acid	Phenylalanine & tyrosine metabolism	tyrosine	0.083	0.240	0.174	0.017
Amino acid	Phenylalanine & tyrosine metabolism	3-(4-hydroxyphenyl)lactate	0.403	0.372	0.692	0.067
Amino acid	Phenylalanine & tyrosine metabolism	4-hydroxyphenylpyruvate	0.353	0.843	0.570	0.085
Amino acid	Phenylalanine & tyrosine metabolism	4-hydroxyphenylacetate	0.223	0.203	0.327	0.459
Amino acid	Phenylalanine & tyrosine metabolism	3-hydroxyphenylacetate	0.252	0.812	0.341	0.846
Amino acid	Phenylalanine & tyrosine metabolism	3-methoxytyrosine	0.919	0.761	0.861	0.739
Amino acid	Phenylalanine & tyrosine metabolism	phenylacetylglutamine	0.253	0.980	0.119	0.885
Amino acid	Phenylalanine & tyrosine metabolism	3-phenylpropionate (hydrocinnamate)	0.250	0.004	0.883	0.122
Amino acid	Phenylalanine & tyrosine metabolism	phenol sulfate	0.081	0.153	0.187	0.817
Amino acid	Tryptophan metabolism	kynurenine	0.606	0.590	0.374	0.230
Amino acid	Tryptophan metabolism	tryptophan	0.039	0.860	0.557	0.035
Amino acid	Tryptophan metabolism	indolelactate	0.219	0.748	0.730	0.546
Amino acid	Tryptophan metabolism	indoleacetate	0.846	0.336	0.202	0.513
Amino acid	Tryptophan metabolism	tryptophan betaine	0.331	0.597	0.848	0.116
Amino acid	Tryptophan metabolism	serotonin (5HT)	0.769	0.279	0.263	0.565
Amino acid	Tryptophan metabolism	C-glycosyltryptophan*	0.179	0.424	0.602	0.686
Amino acid	Tryptophan metabolism	3-indoxyl sulfate	0.014	0.693	0.208	0.043
Amino acid	Tryptophan metabolism	indolepropionate	0.070	0.074	0.459	0.709
Amino acid	Valine, leucine and isoleucine metabolism	3-methyl-2-oxobutyrate	0.030	0.006	0.146	0.001
Amino acid	Valine, leucine and isoleucine metabolism	3-methyl-2-oxovalerate	0.457	0.053	0.136	0.015
Amino acid	Valine, leucine and isoleucine metabolism	beta-hydroxyisovalerate	0.127	0.639	0.738	0.201

Amino acid	Valine, leucine and isoleucine metabolism	alpha-hydroxyisocaproate	0.917	0.004	0.009	0.042
Amino acid	Valine, leucine and isoleucine metabolism	isoleucine	0.805	0.890	0.156	0.251
Amino acid	Valine, leucine and isoleucine metabolism	leucine	0.187	0.883	0.591	0.194
Amino acid	Valine, leucine and isoleucine metabolism	valine	0.621	0.946	0.712	0.701
Amino acid	Valine, leucine and isoleucine metabolism	2-hydroxyisobutyrate	0.502	0.896	0.169	0.693
Amino acid	Valine, leucine and isoleucine metabolism	3-hydroxyisobutyrate	0.066	0.719	0.234	0.396
Amino acid	Valine, leucine and isoleucine metabolism	4-methyl-2-oxopentanoate	0.347	0.046	0.336	0.002
Amino acid	Valine, leucine and isoleucine metabolism	alpha-hydroxyisovalerate	0.304	0.890	0.347	0.336
Amino acid	Valine, leucine and isoleucine metabolism	isobutyrylcarnitine	0.286	0.945	0.195	0.564
Amino acid	Valine, leucine and isoleucine metabolism	2-hydroxy-3-methylvalerate	0.272	0.261	0.028	0.021
Amino acid	Valine, leucine and isoleucine metabolism	2-methylbutyroylcarnitine	0.050	0.261	0.380	0.818
Amino acid	Valine, leucine and isoleucine metabolism	isovalerylcarnitine	0.724	0.688	0.854	0.222
Amino acid	Valine, leucine and isoleucine metabolism	hydroxyisovaleroyl carnitine	0.005	0.297	0.072	0.113
Amino acid	Valine, leucine and isoleucine metabolism	tiglyl carnitine	0.007	0.465	0.127	0.884
Amino acid	Cysteine, methionine, SAM, taurine metabolism	cysteine	0.093	0.876	0.872	0.259
Amino acid	Cysteine, methionine, SAM, taurine metabolism	S-methylcysteine	0.870	0.398	0.173	0.073
Amino acid	Cysteine, methionine, SAM, taurine metabolism	cystine	0.782	0.779	0.591	0.478
Amino acid	Cysteine, methionine, SAM, taurine metabolism	N-formylmethionine	0.002	0.906	0.150	0.024
Amino acid	Cysteine, methionine, SAM, taurine metabolism	methionine	0.186	0.227	0.830	0.034
Amino acid	Cysteine, methionine, SAM, taurine metabolism	N-acetylmethionine	0.063	0.646	0.720	0.053
Amino acid	Cysteine, methionine, SAM, taurine metabolism	2-hydroxybutyrate (AHB)	0.050	0.434	0.054	0.068
Amino acid	Urea cycle; arginine-, proline-, metabolism	symmetric dimethylarginine (SDMA)	0.751	0.513	0.618	0.883
Amino acid	Urea cycle; arginine-, proline-, metabolism	arginine	0.580	0.262	0.693	0.022
Amino acid	Urea cycle; arginine-, proline-, metabolism	ornithine	0.952	0.925	0.605	0.658
Amino acid	Urea cycle; arginine-, proline-, metabolism	urea	0.027	0.774	0.156	0.067
Amino acid	Urea cycle; arginine-, proline-, metabolism	proline	0.797	0.426	0.240	0.132
Amino acid	Urea cycle; arginine-, proline-, metabolism	citrulline	0.966	0.993	0.539	0.372

Amino acid	Urea cycle; arginine-, proline-, metabolism	N-acetylornithine	0.258	0.195	0.343	0.589
Amino acid	Urea cycle; arginine-, proline-, metabolism	N-methyl proline	0.757	0.630	0.778	0.970
Amino acid	Urea cycle; arginine-, proline-, metabolism	trans-4-hydroxyproline	0.084	0.184	0.665	0.485
Amino acid	Creatine metabolism	creatine	0.801	0.282	0.759	0.055
Amino acid	Creatine metabolism	creatinine	0.041	0.310	0.025	0.754
Amino acid	Butanoate metabolism	2-aminobutyrate	0.243	0.841	0.410	0.645
Amino acid	Guanidino and acetamido metabolism	4-acetamidobutanoate	0.209	0.292	0.608	0.328
Amino acid	Glutathione metabolism	5-oxoproline	0.136	0.628	0.138	0.190
Amino acid	Glutathione metabolism	glutathione, oxidized (GSSG)	0.506	0.688	0.992	0.493
Peptide	Dipeptide	aspartylphenylalanine	nd	nd	nd	nd
Peptide	Dipeptide	pro-hydroxy-pro	0.282	0.306	0.568	0.354
Peptide	Dipeptide	phenylalanylphenylalanine	0.073	0.160	0.627	0.270
Peptide	Dipeptide	cyclo(leu-pro)	0.993	0.291	0.053	0.640
Peptide	gamma-glutamyl	gamma-glutamylvaline	0.968	0.649	0.605	0.843
Peptide	gamma-glutamyl	gamma-glutamylleucine	0.605	0.757	0.160	0.502
Peptide	gamma-glutamyl	gamma-glutamylisoleucine*	0.901	0.465	0.222	0.885
Peptide	gamma-glutamyl	gamma-glutamylmethionine	0.898	0.888	0.402	0.590
Peptide	gamma-glutamyl	gamma-glutamylglutamine	0.607	0.389	0.136	0.547
Peptide	gamma-glutamyl	gamma-glutamylphenylalanine	0.938	0.162	0.929	0.009
Peptide	gamma-glutamyl	gamma-glutamyltyrosine	0.760	0.431	0.339	0.275
Peptide	gamma-glutamyl	gamma-glutamylalanine	0.689	0.317	0.840	0.488
Peptide	Polypeptide	bradykinin	0.016	0.252	#DIV/0!	0.351
Peptide	Polypeptide	bradykinin, hydroxy-pro(3)	1.000	0.252	#DIV/0!	0.351
Carbohydrate	Aminosugars metabolism	erythronate*	0.025	0.846	0.058	0.308
Carbohydrate	Aminosugars metabolism	fucose	0.421	0.324	0.655	0.947
Carbohydrate	Fructose, mannose, galactose, starch, and sucrose metabolism	fructose	0.770	0.387	0.229	0.495
Carbohydrate	Fructose, mannose, galactose, starch, and sucrose metabolism	maltose	0.406	0.428	0.381	0.276

Carbohydrate	Fructose, mannose, galactose, starch, and sucrose metabolism	mannose	0.734	0.880	0.934	0.673
Carbohydrate	Fructose, mannose, galactose, starch, and sucrose metabolism	maltotriose	0.422	0.700	0.351	0.897
Carbohydrate	Fructose, mannose, galactose, starch, and sucrose metabolism	maltotetraose	0.445	0.636	0.677	0.365
Carbohydrate	Fructose, mannose, galactose, starch, and sucrose metabolism	methyl-beta-glucopyranoside	0.258	0.285	0.287	0.282
Carbohydrate	Glycolysis, gluconeogenesis, pyruvate metabolism	1,5-anhydroglucitol (1,5-AG)	0.507	0.965	0.211	0.488
Carbohydrate	Glycolysis, gluconeogenesis, pyruvate metabolism	glycerate	0.342	0.220	0.119	0.441
Carbohydrate	Glycolysis, gluconeogenesis, pyruvate metabolism	glucose	0.406	0.709	0.369	0.734
Carbohydrate	Glycolysis, gluconeogenesis, pyruvate metabolism	1,6-anhydroglucose	0.281	0.179	0.803	0.988
Carbohydrate	Glycolysis, gluconeogenesis, pyruvate metabolism	3-phosphoglycerate	0.578	0.070	0.258	0.126
Carbohydrate	Glycolysis, gluconeogenesis, pyruvate metabolism	pyruvate	0.744	0.313	0.846	0.727
Carbohydrate	Glycolysis, gluconeogenesis, pyruvate metabolism	lactate	0.715	0.856	0.846	0.930
Carbohydrate	Glycolysis, gluconeogenesis, pyruvate metabolism	glucuronate	0.875	0.149	0.906	0.251
Carbohydrate	Glyoxylate and dicarboxylate metabolism	oxalate (ethanedioate)	0.914	0.858	0.288	0.499
Carbohydrate	Nucleotide sugars, pentose metabolism	arabitol	0.135	0.047	0.317	0.912
Carbohydrate	Nucleotide sugars, pentose metabolism	ribitol	0.100	0.699	0.717	0.569
Carbohydrate	Nucleotide sugars, pentose metabolism	threitol	0.271	0.496	0.725	0.498
Carbohydrate	Nucleotide sugars, pentose metabolism	arabinose	0.865	0.869	0.306	0.334
Carbohydrate	Nucleotide sugars, pentose metabolism	xylose	0.779	0.287	0.456	0.650
Carbohydrate	Advanced glycation end-product	erythrulose	0.661	0.291	0.915	0.470
Energy	Krebs cycle	citrate	0.162	0.485	0.057	0.940
Energy	Krebs cycle	alpha-ketoglutarate	0.390	0.677	0.860	0.318
Energy	Krebs cycle	succinate	0.010	0.901	0.490	0.026
Energy	Krebs cycle	succinylcarnitine	0.233	0.783	0.200	0.287
Energy	Krebs cycle	fumarate	0.039	0.867	0.181	0.554
Energy	Krebs cycle	malate	0.027	0.785	0.080	0.337
Energy	Oxidative phosphorylation	acetylphosphate	0.886	0.139	0.700	0.198

Energy	Oxidative phosphorylation	phosphate	0.428	0.062	0.095	0.290
Energy	Oxidative phosphorylation	pyrophosphate (PPi)	0.939	0.598	0.476	0.176
Lipid	Essential fatty acid	linoleate (18:2n6)	0.003	0.358	0.012	0.004
Lipid	Essential fatty acid	linolenate [alpha or gamma; (18:3n3 or 6)]	0.007	0.490	0.008	0.000
Lipid	Essential fatty acid	dihomo-linolenate (20:3n3 or n6)	0.000	0.009	0.139	0.066
Lipid	Essential fatty acid	eicosapentaenoate (EPA; 20:5n3)	0.080	0.066	0.049	0.022
Lipid	Essential fatty acid	docosapentaenoate (n3 DPA; 22:5n3)	0.008	0.169	0.001	0.002
Lipid	Essential fatty acid	docosahexaenoate (DHA; 22:6n3)	0.031	0.242	0.033	0.060
Lipid	Short chain fatty acid	valerate	0.700	0.534	0.673	0.624
Lipid	Medium chain fatty acid	caproate (6:0)	0.833	0.070	0.009	0.659
Lipid	Medium chain fatty acid	heptanoate (7:0)	0.176	0.987	0.396	0.107
Lipid	Medium chain fatty acid	caprylate (8:0)	0.518	0.176	0.100	0.566
Lipid	Medium chain fatty acid	pelargonate (9:0)	0.541	0.172	0.215	0.190
Lipid	Medium chain fatty acid	caprate (10:0)	0.651	0.034	0.035	0.848
Lipid	Medium chain fatty acid	undecanoate (11:0)	0.320	0.679	0.930	0.588
Lipid	Medium chain fatty acid	10-undecenoate (11:1n1)	0.396	0.155	0.032	0.571
Lipid	Medium chain fatty acid	laurate (12:0)	0.986	0.140	0.024	0.992
Lipid	Medium chain fatty acid	5-dodecenoate (12:1n7)	0.048	0.650	0.050	0.009
Lipid	Long chain fatty acid	myristate (14:0)	0.023	0.038	0.002	0.003
Lipid	Long chain fatty acid	myristoleate (14:1n5)	0.055	0.942	0.037	0.110
Lipid	Long chain fatty acid	pentadecanoate (15:0)	0.032	0.000	0.002	0.000
Lipid	Long chain fatty acid	palmitate (16:0)	0.000	0.014	0.000	0.000
Lipid	Long chain fatty acid	palmitoleate (16:1n7)	0.057	0.577	0.083	0.003
Lipid	Long chain fatty acid	margarate (17:0)	0.000	0.035	0.001	0.000
Lipid	Long chain fatty acid	10-heptadecenoate (17:1n7)	0.015	0.130	0.008	0.001
Lipid	Long chain fatty acid	stearate (18:0)	0.000	0.038	0.002	0.000
Lipid	Long chain fatty acid	oleate (18:1n9)	0.018	0.617	0.138	0.016

Lipid	Long chain fatty acid	cis-vaccenate (18:1n7)	0.009	0.894	0.016	0.021
Lipid	Long chain fatty acid	stearidonate (18:4n3)	0.020	0.461	0.001	0.586
Lipid	Long chain fatty acid	nonadecanoate (19:0)	0.000	0.052	0.024	0.001
Lipid	Long chain fatty acid	10-nonadecenoate (19:1n9)	0.007	0.075	0.002	0.001
Lipid	Long chain fatty acid	eicosenoate (20:1n9 or 11)	0.013	0.081	0.006	0.002
Lipid	Long chain fatty acid	dihomo-linoleate (20:2n6)	0.009	0.043	0.001	0.000
Lipid	Long chain fatty acid	arachidonate (20:4n6)	0.002	0.029	0.017	0.000
Lipid	Long chain fatty acid	adrenate (22:4n6)	0.004	0.009	0.002	0.004
Lipid	Fatty acid, ester	n-Butyl Oleate	0.126	0.296	0.165	0.786
Lipid	Fatty acid, monohydroxy	2-hydroxyoctanoate	0.497	0.716	0.362	0.557
Lipid	Fatty acid, monohydroxy	3-hydroxyoctanoate	0.008	0.227	0.245	0.348
Lipid	Fatty acid, monohydroxy	3-hydroxydecanoate	0.006	0.844	0.041	0.014
Lipid	Fatty acid, monohydroxy	2-hydroxystearate	0.000	0.001	0.000	0.002
Lipid	Fatty acid, monohydroxy	2-hydroxypalmitate	0.001	0.000	0.000	0.002
Lipid	Fatty acid, monohydroxy	13-HODE + 9-HODE	0.057	0.246	0.003	0.032
Lipid	Fatty acid, dicarboxylate	azelate (nonanedioate)	0.085	0.166	0.518	0.733
Lipid	Fatty acid, dicarboxylate	dodecanedioate	0.154	0.317	0.058	0.309
Lipid	Fatty acid, dicarboxylate	tetradecanedioate	0.030	0.615	0.010	0.048
Lipid	Fatty acid, dicarboxylate	hexadecanedioate	0.039	0.561	0.012	0.030
Lipid	Fatty acid, dicarboxylate	octadecanedioate	0.119	0.850	0.222	0.550
Lipid	Fatty acid, dicarboxylate	3-carboxy-4-methyl-5-propyl-2-furanpropanoate (CMPF)	0.153	0.161	0.837	0.707
Lipid	Fatty acid, branched	13-methylmyristic acid	0.004	0.014	0.012	0.004
Lipid	Fatty acid, branched	15-methylpalmitate (isobar with 2-methylpalmitate)	0.005	0.001	0.011	0.000
Lipid	Fatty acid, branched	17-methylstearate	0.026	0.178	0.319	0.001
Lipid	Fatty acid metabolism (also BCAA metabolism)	propionylcarnitine	0.291	0.693	0.827	0.417
Lipid	Fatty acid metabolism (also BCAA metabolism)	butyrylcarnitine	0.020	0.289	0.030	0.463
Lipid	Fatty acid metabolism	valerylcarnitine	0.300	0.340	0.484	0.462

Lipid	Fatty acid metabolism	isovalerate	0.952	0.013	0.766	0.147
Lipid	Carnitine metabolism	deoxycarnitine	0.143	0.241	0.265	0.407
Lipid	Carnitine metabolism	carnitine	0.358	0.499	0.461	0.253
Lipid	Carnitine metabolism	3-dehydrocarnitine*	0.708	0.978	0.464	0.837
Lipid	Carnitine metabolism	acetylcarnitine	0.021	0.436	0.092	0.477
Lipid	Carnitine metabolism	hexanoylcarnitine	0.000	0.259	0.015	0.991
Lipid	Carnitine metabolism	octanoylcarnitine	0.002	0.130	0.039	0.304
Lipid	Carnitine metabolism	decanoylcarnitine	0.013	0.191	0.069	0.411
Lipid	Carnitine metabolism	cis-4-decenoyl carnitine	0.027	0.134	0.110	0.321
Lipid	Carnitine metabolism	laurylcarnitine	0.102	0.299	0.040	0.255
Lipid	Carnitine metabolism	palmitoylcarnitine	0.605	0.325	0.208	0.010
Lipid	Carnitine metabolism	stearoylcarnitine	0.941	0.627	0.440	0.153
Lipid	Carnitine metabolism	oleoylcarnitine	0.267	0.215	0.187	0.022
Lipid	Bile acid metabolism	cholate	0.646	0.369	0.980	0.600
Lipid	Bile acid metabolism	glycocholate	0.101	0.908	0.152	0.298
Lipid	Bile acid metabolism	chenodeoxycholate	0.083	0.491	0.631	0.056
Lipid	Bile acid metabolism	deoxycholate	0.440	0.795	0.702	0.782
Lipid	Bile acid metabolism	glycodeoxycholate	0.390	0.780	0.988	0.305
Lipid	Bile acid metabolism	glycochenodeoxycholate	0.584	0.633	0.578	0.490
Lipid	Bile acid metabolism	glycolithocholate sulfate*	0.657	0.777	0.281	0.049
Lipid	Bile acid metabolism	taurolithocholate 3-sulfate	0.683	0.154	0.355	0.775
Lipid	Bile acid metabolism	glycocholenate sulfate*	0.982	0.053	0.556	0.027
Lipid	Bile acid metabolism	taurocholenate sulfate*	0.391	0.923	0.979	0.298
Lipid	Bile acid metabolism	glycoursodeoxycholate	0.556	0.833	0.710	0.612
Lipid	Glycerolipid metabolism	phosphoethanolamine	0.738	0.150	0.570	0.094
Lipid	Glycerolipid metabolism	choline	0.924	0.351	0.732	0.401
Lipid	Glycerolipid metabolism	glycerol 3-phosphate (G3P)	0.056	0.151	0.317	0.040

Lipid	Glycerolipid metabolism	glycerophosphorylcholine (GPC)	0.981	0.484	0.446	0.881
Lipid	Glycerolipid metabolism	trimethylamine N-oxide	nd	nd	nd	nd
Lipid	Inositol metabolism	myo-inositol	0.493	0.153	0.838	0.511
Lipid	Inositol metabolism	chiro-inositol	0.356	0.683	0.988	0.682
Lipid	Ketone bodies	3-hydroxybutyrate (BHBA)	0.075	0.861	0.188	0.325
Lipid	Lysolipid	1-palmitoylglycerophosphoethanolamine	0.260	0.007	0.787	0.004
Lipid	Lysolipid	2-palmitoylglycerophosphoethanolamine*	0.114	0.004	0.745	0.000
Lipid	Lysolipid	1-stearoylglycerophosphoethanolamine	0.667	0.299	0.716	0.270
Lipid	Lysolipid	1-oleoylglycerophosphoethanolamine	0.029	0.006	0.350	0.001
Lipid	Lysolipid	2-oleoylglycerophosphoethanolamine*	0.227	0.060	0.580	0.023
Lipid	Lysolipid	1-linoleoylglycerophosphoethanolamine*	0.550	0.976	0.157	1.000
Lipid	Lysolipid	2-linoleoylglycerophosphoethanolamine*	0.572	0.006	0.491	0.044
Lipid	Lysolipid	1-arachidonoylglycerophosphoethanolamine*	0.300	0.002	0.032	0.002
Lipid	Lysolipid	2-arachidonoylglycerophosphoethanolamine*	0.749	0.959	0.057	0.545
Lipid	Lysolipid	2-docosahexaenoylglycerophosphoethanolamine*	0.426	0.312	0.005	0.204
Lipid	Lysolipid	1-myristoylglycerophosphocholine	0.615	0.903	0.031	0.387
Lipid	Lysolipid	2-myristoylglycerophosphocholine*	0.811	0.797	0.134	0.169
Lipid	Lysolipid	1-pentadecanoylglycerophosphocholine*	0.548	0.562	0.089	0.445
Lipid	Lysolipid	1-palmitoylglycerophosphocholine	0.650	0.914	0.046	0.552
Lipid	Lysolipid	2-palmitoylglycerophosphocholine*	0.967	0.974	0.136	0.502
Lipid	Lysolipid	1-palmitoleoylglycerophosphocholine*	0.064	0.492	0.069	0.791
Lipid	Lysolipid	2-palmitoleoylglycerophosphocholine*	0.181	0.817	0.053	0.875
Lipid	Lysolipid	1-heptadecanoylglycerophosphocholine	0.710	0.514	0.110	0.675
Lipid	Lysolipid	1-stearoylglycerophosphocholine	0.609	0.797	0.315	0.797
Lipid	Lysolipid	2-stearoylglycerophosphocholine*	0.297	0.853	0.068	0.979
Lipid	Lysolipid	1-oleoylglycerophosphocholine	0.173	0.683	0.037	0.937
Lipid	Lysolipid	2-oleoylglycerophosphocholine*	0.764	0.304	0.030	0.195

Lipid	Lysolipid	1-linoleoylglycerophosphocholine	0.401	0.966	0.022	0.991
Lipid	Lysolipid	2-linoleoylglycerophosphocholine*	0.207	0.395	0.046	0.735
Lipid	Lysolipid	1-eicosadienoylglycerophosphocholine*	0.187	0.832	0.018	0.736
Lipid	Lysolipid	1-eicosatrienoylglycerophosphocholine*	0.411	0.376	0.041	0.384
Lipid	Lysolipid	1-arachidonoylglycerophosphocholine*	0.232	0.822	0.051	0.813
Lipid	Lysolipid	2-arachidonoylglycerophosphocholine*	0.474	0.963	0.002	0.599
Lipid	Lysolipid	1-docosapentaenoylglycerophosphocholine*	0.520	0.948	0.026	0.587
Lipid	Lysolipid	1-docosahexaenoylglycerophosphocholine*	0.035	0.485	0.035	0.905
Lipid	Lysolipid	2-docosahexaenoylglycerophosphocholine*	0.611	0.339	0.188	0.035
Lipid	Lysolipid	1-stearoylglycerophosphoinositol	0.088	0.001	0.045	0.003
Lipid	Lysolipid	1-arachidonoylglycerophosphoinositol*	0.105	0.000	0.003	0.003
Lipid	Lysolipid	1-palmitoylplasmenylethanolamine*	0.248	0.058	0.052	0.061
Lipid	Sphingolipid	sphingosine	0.184	0.477	0.904	0.532
Lipid	Sphingolipid	palmitoyl sphingomyelin	0.382	0.407	0.289	0.379
Lipid	Sphingolipid	stearoyl sphingomyelin	0.366	0.668	0.394	0.415
Lipid	Sterol/Steroid	lathosterol	0.256	0.354	0.704	0.945
Lipid	Sterol/Steroid	squalene	nd	nd	nd	nd
Lipid	Sterol/Steroid	cholesterol	0.167	0.341	0.040	0.377
Lipid	Sterol/Steroid	dehydroisoandrosterone sulfate (DHEA-S)	0.550	0.552	0.035	0.001
Lipid	Sterol/Steroid	epiandrosterone sulfate	0.713	0.200	0.210	0.019
Lipid	Sterol/Steroid	androsterone sulfate	0.888	0.471	0.193	0.006
Lipid	Sterol/Steroid	cortisol	0.365	0.376	0.478	0.722
Lipid	Sterol/Steroid	cortisone	0.864	0.195	0.717	0.214
Lipid	Sterol/Steroid	beta-sitosterol	0.574	0.883	0.880	0.789
Lipid	Sterol/Steroid	7-alpha-hydroxy-3-oxo-4-cholestenoate (7-Hoca)	0.035	0.989	0.040	0.038
Lipid	Sterol/Steroid	4-androsten-3beta,17beta-diol disulfate 1*	0.591	0.661	0.298	0.035
Lipid	Sterol/Steroid	4-androsten-3beta,17beta-diol disulfate 2*	0.808	0.367	0.458	0.003

Lipid	Sterol/Steroid	5alpha-androstan-3beta,17beta-diol disulfate	0.430	0.391	0.136	0.199
Lipid	Sterol/Steroid	5alpha-pregnan-3beta,20alpha-diol disulfate	0.469	0.209	0.982	0.396
Lipid	Sterol/Steroid	pregnen-diol disulfate*	0.832	0.911	0.679	0.045
Lipid	Sterol/Steroid	pregn steroid monosulfate*	0.599	0.429	0.157	0.005
Lipid	Sterol/Steroid	andro steroid monosulfate 1*	0.126	0.416	0.105	0.270
Lipid	Sterol/Steroid	andro steroid monosulfate 2*	0.742	0.313	0.647	0.042
Lipid	Sterol/Steroid	21-hydroxypregnenolone disulfate	0.550	0.410	0.432	0.001
Lipid	Sterol/Steroid	pregnenolone sulfate	0.788	0.027	0.976	0.003
Nucleotide	Purine metabolism, (hypo)xanthine/inosine containing	xanthine	0.486	0.456	0.393	0.337
Nucleotide	Purine metabolism, (hypo)xanthine/inosine containing	hypoxanthine	0.973	0.033	0.564	0.264
Nucleotide	Purine metabolism, adenine containing	N1-methyladenosine	0.224	0.317	0.731	0.066
Nucleotide	Purine metabolism, adenine containing	adenosine 5'-monophosphate (AMP)	0.954	0.615	0.525	0.713
Nucleotide	Purine metabolism, adenine containing	adenosine 5'-diphosphate (ADP)	0.543	0.235	0.558	0.094
Nucleotide	Purine metabolism, guanine containing	N1-methylguanosine	0.287	0.128	0.140	0.154
Nucleotide	Purine metabolism, guanine containing	N2,N2-dimethylguanosine	0.909	0.871	0.002	0.187
Nucleotide	Purine metabolism, guanine containing	N6-carbamoylthreonyladenosine	0.070	0.029	0.154	0.198
Nucleotide	Purine metabolism, urate metabolism	urate	0.010	0.852	0.025	0.001
Nucleotide	Pyrimidine metabolism, cytidine containing	N4-acetylcytidine	0.820	0.763	0.269	0.246
Nucleotide	Pyrimidine metabolism, uracil containing	uridine	0.424	0.013	0.093	0.066
Nucleotide	Pyrimidine metabolism, uracil containing	pseudouridine	0.001	0.337	0.002	0.000
Nucleotide	Pyrimidine metabolism, uracil containing	5-methyluridine (ribothymidine)	0.170	0.018	0.012	0.034
Nucleotide	Purine and pyrimidine metabolism	methylphosphate	0.102	0.643	0.411	0.202
Cofactors and vitamins	Ascorbate and aldarate metabolism	threonate	0.182	0.467	0.425	0.078
Cofactors and vitamins	Hemoglobin and porphyrin metabolism	heme*	0.391	0.155	0.828	0.648
Cofactors and vitamins	Hemoglobin and porphyrin metabolism	L-urobilin	0.253	0.945	0.946	0.072
Cofactors and vitamins	Hemoglobin and porphyrin metabolism	bilirubin (Z,Z)	0.133	0.195	0.851	0.658

Cofactors and vitamins	Hemoglobin and porphyrin metabolism	bilirubin (E,E)*	0.003	0.166	0.195	0.091
Cofactors and vitamins	Hemoglobin and porphyrin metabolism	bilirubin (E,Z or Z,E)*	0.002	0.001	0.389	0.776
Cofactors and vitamins	Hemoglobin and porphyrin metabolism	biliverdin	0.100	0.161	0.005	0.053
Cofactors and vitamins	Nicotinate and nicotinamide metabolism	nicotinamide	0.945	0.178	0.365	0.506
Cofactors and vitamins	Nicotinate and nicotinamide metabolism	adenosine 5'diphosphoribose	0.460	0.051	0.490	0.011
Cofactors and vitamins	Nicotinate and nicotinamide metabolism	trigonelline (N'-methylnicotinate)	0.242	0.833	0.507	0.549
Cofactors and vitamins	Pantothenate and CoA metabolism	pantothenate	0.597	0.524	0.717	0.476
Cofactors and vitamins	Riboflavin metabolism	flavin adenine dinucleotide (FAD)	0.041	0.004	0.126	0.022
Cofactors and vitamins	Tocopherol metabolism	alpha-tocopherol	0.210	0.109	0.226	0.208
Cofactors and vitamins	Tocopherol metabolism	gamma-CEHC	0.676	0.841	0.642	0.963
Cofactors and vitamins	Vitamin B6 metabolism	pyridoxate	0.029	0.537	0.086	0.749
Xenobiotics	Benzoate metabolism	hippurate	0.453	0.148	0.512	0.412
Xenobiotics	Benzoate metabolism	2-hydroxyhippurate (salicylurate)	0.409	0.833	0.905	0.007
Xenobiotics	Benzoate metabolism	3-hydroxyhippurate	0.643	0.681	0.754	0.810
Xenobiotics	Benzoate metabolism	catechol sulfate	0.222	0.937	0.361	0.639
Xenobiotics	Benzoate metabolism	benzoate	0.098	0.697	0.064	0.436
Xenobiotics	Benzoate metabolism	methyl-4-hydroxybenzoate	0.845	0.640	0.206	0.427
Xenobiotics	Benzoate metabolism	4-ethylphenylsulfate	0.845	0.817	0.790	0.633
Xenobiotics	Benzoate metabolism	4-vinylphenol sulfate	0.215	0.042	0.909	0.450
Xenobiotics	Chemical	glycolate (hydroxyacetate)	0.049	0.724	0.183	0.206
Xenobiotics	Chemical	iminodiacetate (IDA)	0.091	0.130	0.088	0.173
Xenobiotics	Chemical	glycerol 2-phosphate	0.007	0.997	0.164	0.090
Xenobiotics	Chemical	2-ethylhexanoate	0.241	0.632	0.280	0.971
Xenobiotics	Chemical	diisopropanolamine	nd	nd	nd	nd

Xenobiotics	Drug	codeine	nd	nd	nd	nd
Xenobiotics	Drug	4-acetaminophen sulfate	0.891	0.672	0.381	0.171
Xenobiotics	Drug	4-acetamidophenol	0.298	0.252	0.341	0.351
Xenobiotics	Drug	p-acetamidophenylglucuronide	0.535	0.252	0.341	0.176
Xenobiotics	Drug	2-hydroxyacetaminophen sulfate*	0.994	0.643	0.395	0.671
Xenobiotics	Drug	2-methoxyacetaminophen sulfate*	0.589	0.252	0.341	0.351
Xenobiotics	Drug	3-(cystein-S-yl)acetaminophen*	0.382	0.252	0.341	0.351
Xenobiotics	Drug	2-methoxyacetaminophen glucuronide*	0.458	0.252	0.341	0.351
Xenobiotics	Drug	ibuprofen	0.295	0.670	0.872	0.377
Xenobiotics	Drug	naproxen	nd	nd	nd	nd
Xenobiotics	Drug	desmethylnaproxen sulfate*	nd	nd	nd	nd
Xenobiotics	Drug	pivaloylcarnitine	0.480	0.196	0.318	0.341
Xenobiotics	Drug	pseudoephedrine	nd	nd	nd	nd
Xenobiotics	Drug	quinine	0.797	0.518	0.617	0.320
Xenobiotics	EDTA	EDTA	0.026	0.018	0.139	0.025
Xenobiotics	Food component/Plant	saccharin	0.088	0.364	0.172	0.114
Xenobiotics	Food component/Plant	quinate	0.103	0.680	0.056	0.471
Xenobiotics	Food component/Plant	piperine	0.161	0.265	0.179	0.382
Xenobiotics	Food component/Plant	thymol sulfate	0.995	0.206	0.187	0.046
Xenobiotics	Food component/Plant	stachydrine	0.542	0.146	0.111	0.919
Xenobiotics	Food component/Plant	homostachydrine*	0.464	0.476	0.232	0.149
Xenobiotics	Food component/Plant	cinnamoylglycine	0.641	0.206	0.485	0.077
Xenobiotics	Xanthine metabolism	caffeine	0.561	0.765	0.959	0.110
Xenobiotics	Xanthine metabolism	paraxanthine	0.509	0.900	0.298	0.287
Xenobiotics	Xanthine metabolism	theobromine	0.577	0.593	0.247	0.216
Xenobiotics	Xanthine metabolism	theophylline	0.139	0.172	0.700	0.715
Xenobiotics	Xanthine metabolism	1-methylurate	0.473	0.146	0.391	0.861

Xenobiotics	Xanthine metabolism	1,7-dimethylurate	0.323	0.969	0.733	0.161
Xenobiotics	Xanthine metabolism	1-methylxanthine	0.568	0.027	0.271	0.033
Xenobiotics	Xanthine metabolism	3-methylxanthine	0.825	0.107	0.523	0.057
Xenobiotics	Xanthine metabolism	7-methylxanthine	0.501	0.678	0.573	0.405
Xenobiotics	Tobacco metabolite	cotinine	0.252	0.252	nd	0.351
Xenobiotics	Sugar, sugar substitute, starch	erythritol	0.138	0.825	0.083	0.580

[1] T-tests were used to calculate P values for the likelihood of significant differences between and within genotypes pre and post intervention. Yellow denotes genotype 1> genotype 2, orange genotype 2>genotype 1, blue pre>post, red post>pre
